# Unlocking the relationships among population structure, plant architecture, growing season, and environmental adaptation in Henan wheat cultivars

**DOI:** 10.1186/s12870-020-02674-z

**Published:** 2020-10-12

**Authors:** Jian Yang, Yanjie Zhou, Weiguo Hu, Yu’e Zhang, Yong Zhou, Yongxing Chen, Xicheng Wang, Hong Zhao, Tingjie Cao, Zhiyong Liu

**Affiliations:** 1 Wheat Research Institute, Henan Academy of Agricultural Sciences, Zhengzhou, 450002 Henan China; 2 Henan Academy of Crop Molecular Breeding, Henan Academy of Agricultural Sciences, Zhengzhou, 450002 China; 3 Center for Desert Agriculture, Biological and Environmental Sciences & Engineering Division (BESE), King Abdullah University of Science and Technology (KAUST), Thuwal, 23955-6900 Saudi Arabia; 4 State Key Laboratory of Plant Cell and Chromosome Engineering, Institute of Genetics and Developmental Biology, Chinese Academy of Sciences, Beijing, 100101 China

**Keywords:** Elite wheat cultivars, Population structure, Yield, GWAS, Selective sweep

## Abstract

**Background:**

Ecological environments shape plant architecture and alter the growing season, which provides the basis for wheat genetic improvement. Therefore, understanding the genetic basis of grain yield and yield-related traits in specific ecological environments is important.

**Results:**

A structured panel of 96 elite wheat cultivars grown in the High-yield zone of Henan province in China was genotyped using an Illumina iSelect 90 K SNP assay. Selection pressure derived from ecological environments of mountain front and plain region provided the initial impetus for population divergence. This determined the dominant traits in two subpopulations (spike number and spike percentage were dominance in subpopulation 2:1; thousand-kernel weight, grain filling rate (GFR), maturity date (MD), and fertility period (FP) were dominance in subpopulation 2:2), which was also consistent with their inheritance from the donor parents. Genome wide association studies identified 107 significant SNPs for 12 yield-related traits and 10 regions were pleiotropic to multiple traits. Especially, GY was co-located with MD/FP, GFR and HD at *QTL-ple5A*, *QTL-ple7A.1* and *QTL-ple7B.1* region. Further selective sweep analysis revealled that regions under selection were around QTLs for these traits. Especially, grain yield (GY) is positively correlated with MD/FP and they were co-located at the *VRN-1A* locus. Besides, a selective sweep signal was detected at *VRN-1B* locus which was only significance to MD/FP.

**Conclusions:**

The results indicated that extensive differential in allele frequency driven by ecological selection has shaped plant architecture and growing season during yield improvement. The QTLs for yield and yield components detected in this study probably be selectively applied in molecular breeding.

## Background

Wheat, maize, and rice are the three most important food crops in the world. With the ongoing increase in the global population, climate change, and reduced availability of arable land, gains in yield of ~ 2% annually and a cumulative increase of 50% in ~ 20 years are required to meet the predicted global demand.

The northwest to centre-east region of Henan province in China, located south of the Yellow and Huai river valleys, is the largest wheat-producing and high-yield area in China. The region contributes one-quarter of the total annual wheat production in China, thus attaining high yields is the core objective of wheat production in the region. The main wheat-growing area is located in the northern subtropical zone, which experiences four distinct seasons, and a transitional zone between the second and third terraces of China. As a consequence, these complex ecological environments enable wheat cultivars with various growing seasons (semi-winter and weak-spring) and plant architecture types to be grown in the region.

In the early twenty-first century, wheat yield in Henan province increased rapidly and remarkable progress was achieved in improving grain yield compared with production in the preceding period (Zhou et al. 2007). A number of cultivars that attain high and stable yields and show adaptability are recommended for cultivation in Henan and are accepted as founder parents. For example, Yumai 2, Zhou 8425B, and Yanshi 4 have been repeatedly utilized as donor parents in different zones to various degrees. Yumai 2 is a weak-winter cultivar with a weak-spring habit, which exhibits strong tillering ability and cold resistance but lower grain weight (Zheng et al. 2011; Gao et al. 2017). Yanshi 4 is a spring wheat cultivar derived from Funo and Mara that produces large spikes and is early maturing, but its tillering ability is weak. The 1B/1R translocation line Zhou 8425B is a high-yielding, strongly disease-resistant wheat cultivar with large spikes and dwarf habit (Gao et al. 2015; Zhao et al. 2008; Li et al. 2006; Wang et al. 2017). Numerous progeny bred from these cultivars inherited desirable characters and were approved for commercial release. Yumai 25, Yumai 41, and Yumai 49 were selected from the cross between 394A and Yumai 2, and inherited the early maturity of Yumai 2. Zhoumai 9 (Yumai 21), which was bred by double-crossing Yumai 2 and Yanshi 4, exhibits high grain weight and semi-dwarfism but shows later maturity, and thus is suitable for planting in central Henan with early sowing. Further pyramiding of Zhou 8425B resulted in a series of Zhoumai-family wheat cultivars (e.g., Zhoumai 13, Zhoumai 16, and Zhoumai 22). In addition, a number of cultivars imported from other regions (e.g., Shaanxi) have been used as parents to shorten the fertility period, introduce disease resistance, improve grain end-use quality, and have contributed to an increase in genetic diversity.

Population genetics based on molecular markers and phenotype analysis are widely used to detect chromosomal regions important in species evolution, to identify genetic variation associated with traits beneficial for human health, growth characteristics of animals, and genomic regions that contribute to important traits [1–6]. One approach is to conduct a genome-wide association study of a genetically diverse panel of natural accessions for quantitative trait locus (QTL) discovery by linkage of genotypes with phenotypes to determine the underlying genetic basis of desirable traits. In particular, it has enabled substantial progress in dissection of pleiotropic QTLs to understand the underlying genetic basis of complex traits [7–10]. An alternative approach is selective sweep analysis, which screens the differentiation in allele frequencies between subpopulations. A selective sweep is the result of a remarkable reduction in variation among nucleotide sequences neighboring mutations beneficial for fitness during domestication or adaptation [11, 12]. The method has been widely applied in plant population genetics to identify signals associated with fruit quality improvement [12], flowering-time divergence among different ecotypes [13], and overwintering habits [14].

Understanding the genetic basis of phenotypic variation among wheat cultivars and discovering the genetic footprint of environmental adaptation in different regions of Henan, and integration of this information in future cultivar development programs, is of considerable importance for continued improvement in wheat yields. To attain this goal, we assembled a panel of elite breeding cultivars representative of the most genetic diversity among modern wheat cultivars grown in the main wheat-producing zone of Henan province for phenotype evaluation. Population genetic analysis was conducted to assess population structure and identify the genomic regions that affect the plant architecture or growing season along with environmental adaptation.

## Results

### Population structure and phenotype between populations

**Fig. 1:**
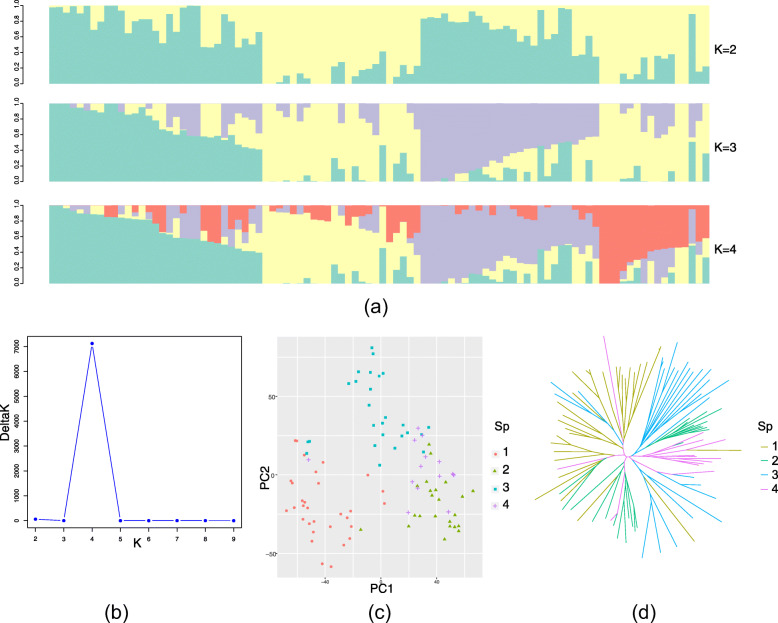
Population structure of association mapping panel of wheat accessions from Henan province based on the iSelect 90 K SNP genotyping. **a** Membership coefficient (Q-value) where each horizontal line represents ranged wheat cultivars (X-axis) and the accessions were partitioned into four subpopulations. Y axis represented the percentage of shared alleles between paired lines. **b** The plot of the scaled mean logarithm of the probability of data likelihood [LnP (D)] (Y-axis) and delta K (ΔK) with K (X-axis) allowed ranging from 2 to 10. **c** Plot of the first two principal components illustrated four subpopulations which assigned in STRUCTURE result. **d** Neighbor-joining phylogenetic tree of 96 wheat cultivars. Colors of branches in the tree indicate matching the population inferior by Sp1 to Sp4

**Fig. 2:**
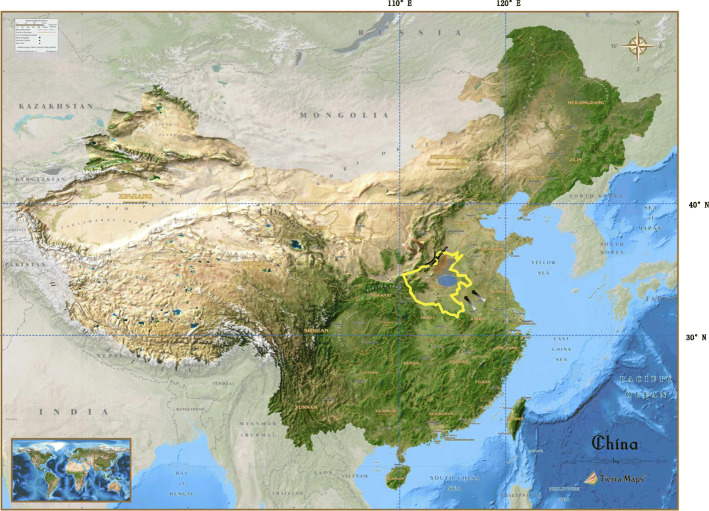
The map of China. The main wheat production areas in Henan are marked in yellow box. The orange area is the Northwest plain of Henan; Blue aera is centre-east plain of Henan; grey is the intermediate region. The Gradient black arrow is the southeast monsoon in summer; Gradient red arrow is northwest monsoon in winter

A total of 81,088 SNP markers were used for assessment of population structure. All cultivars were assessed from *K* = 2 to *K* = 4 (Fig. 1a, Table S1). At *K* = 2, wheat cultivars in subpopulation 2:1 were derived from the donor parents Yumai 2, Yanshi 4, and Shaanxi, and were mostly selected in the northwest to central region of Henan, whereas subpopulation 2:2 exclusively comprised cultivars with the pedigree of Yumai 2, Zhou 8425B, and Yanshi 4 harboring the rye 1RS chromosome arm and were selected in the central region of the southeastern plains (Fig. 2). At *K* = 3, cultivars in subgroup 2:1 were resolved into two subpopulations: subpopulation 3:1 (Sp1) and subpopulation 3:3 (Sp3). The cultivars in subgroup 3:1 were predominantly derived from Yumai 2, Yanshi 4, and Neixiang 82C6, and consisted of lines harboring the normal wheat 1BS chromosome arm, whereas cultivars in subgroup 3:3 comprised mixed donor parents included Yumai 2, Yanshi 4, and cultivars in other regions (such as Shaanxi) and harbored 1B/1R chromosome translocations. At *K* = 4, cultivars in subgroup 2 (*K* = 2) were divided into an additional two subpopulations: subpopulation 4:2 (Sp2) and subpopulation 4:4 (Sp4). The majority of cultivars in Sp4 were second-generation derivatives (Zhoumai 13 and Zhoumai 16) of Yanshi 4 bred by pyramiding the donor parents with Zhou 8425B, whereas cultivars in Sp2 were derived from donor parents in Henan other than Zhou 8425B and Yanshi 4. In addition, cultivars grouped in Sp1 and Sp3 were suitable for growth in northwest-central Henan, whereas cultivars in Sp2 and Sp4 were suitable for cultivation in central-east Henan. The plot of the mean likelihood L(*K*) and variance per *K* value indicated that *K* = 4 was the most likely number of subgroups among the 96 cultivars (Fig. 1b).

Assignment to the four subpopulations superimposed on the results of the PCA analysis was similar to the STRUCTURE results. In the PCA analysis, PC1 separated Sp1 (cultivars harboring the 1BS chromosome arm) from other cultivars with no discrimination of the other three subpopulations (Fig. 1c). The PC2 separated Sp2 and Sp3, and Sp4 was distinguished by PC3 (Fig. S1a).

To investigate phylogenetic relationships among the cultivars, a phylogenetic tree was constructed based on the genotyping data for the 96 cultivars. Cultivars grouped in Sp1 and Sp3 showed a congruent relationship with the results of STRUCTURE and PCA with few exceptions (Fig. 1d). The cultivars grouped in Sp2 were divided into two clusters: one cluster diverged from the other three subpopulations and the second cluster was linked to Sp3 accompanied by the Sp4 cluster.

Number of variable sites, nucleotide diversity (π), and average number of nucleotide differences were highest in Sp3 (35,722, 0.13862, and 11,230.98265, respectively), followed by Sp1 (31,897, 0.12774, and 10,348.97462) and Sp2 (27,206, 0.11351, and 9196.18937), and were lowest in Sp4 (27,684, 0.10994, and 8906.97984) (Table S3).

The mean LD estimates ranged from *r*^2^ = 0.82 (0–0.5 Mb) to *r*^2^ = 0.12 (436–436.5 Mb) (Fig. S1b). The LD score rapidly decayed from 0 to 10 Mb and showed an approximate inflection point of *r*^2^ > 0.6. The LD decay showed a moderate decrease within 10–60 Mb with *r*^2^ ranging from 0.6 to 0.4. The fitted regression intersected the threshold at approximately 30 Mb with average LD decay at *r*^2^ = 0.5.

### Phenotypic trait evaluation and correlation

**Fig. 3:**
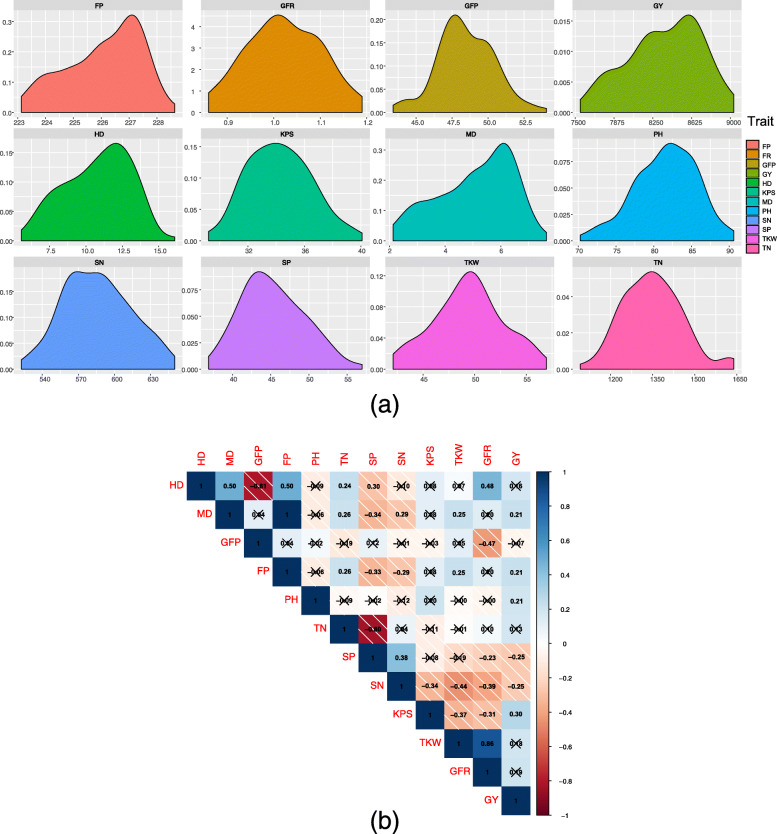
Phenotype description for twelve traits for Henan wheat population. **a** Kernel density of BLUP value of twelve traits  (x-axis: phenotype distribution, y-axis: density). The x-axis showed the BLUPvalue range for each trait. **b** Pairwise correlation of twelve traits. The number showed the Pearson correlation coefficient and the number without cross represented that two traits were significantly correlated (*p* < 0.05)

The phenotype in the two environments was significantly correlated (*p* < 0.01) and the kernel density distribution of phenotype BLUP values showed that all traits exhibited a continuous distribution (Fig. 3a). Broad-sense heritability on the tested 12 traits ranged from 0.53 (TN) to 0.98 (MD and FP). For seven traits (HD, MD, GFP, FP, SN, TKW, and GFR), *H*^2^ was greater than 0.9, whereas *H*^2^ for GY, KPS, TN, PH, and SP was 0.684, 0.656, 0.531, 0.531, and 0.473, respectively (Table 1).

**Table 1:** Phenotype overview in two environments and estimation of broad sense heritability

Trait	2014Anyang				2014Xinxiang				Correlation (**p < 0.01)	Broad Sense Heridity	BLUP			
	min	max	mean	stdev	min	max	mean	stdev			min	max	mean	stdev
HD	7.00	18.00	12.46	2.35	3.50	14.50	8.93	2.44	0.92^**^	0.96	5.55	16.12	10.78	2.26
MD	3.50	10.00	6.65	1.51	0.50	5.50	3.50	1.28	0.97^**^	0.96	2.13	7.64	5.07	1.33
GFP	42.50	54.00	48.28	2.24	43.50	55.00	48.66	2.13	0.88^**^	0.93	43.40	54.06	48.47	1.97
FP	223.50	230.00	226.65	1.51	222.50	227.50	225.50	1.28	0.97^**^	0.96	223.13	228.64	226.07	1.33
TN	67.50	89.50	79.73	4.40	71.00	95.25	83.13	4.70	0.87^**^	0.53	70.26	90.61	81.59	4.10
SP	53.20	128.47	93.28	16.78	56.17	137.33	87.27	16.01	0.33^**^	0.47	72.78	109.29	90.01	7.11
SN	27.66	61.03	41.65	7.67	29.12	72.97	49.36	9.35	0.38^**^	0.92	36.87	56.96	45.32	4.16
PH	30.02	46.27	37.57	3.53	32.90	52.21	40.88	4.02	0.31^**^	0.50	34.84	43.36	39.06	1.86
KPS	21.65	44.10	32.52	4.10	24.80	45.03	35.96	3.88	0.49^**^	0.66	29.22	40.08	34.24	2.26
TKW	40.32	58.86	49.69	4.07	41.34	56.08	49.11	3.34	0.91^**^	0.94	42.08	56.96	49.40	3.39
GFR	0.81	1.22	1.03	0.09	0.83	1.19	1.01	0.08	0.89^**^	0.93	0.86	1.19	1.02	0.08
GY	418.95	628.18	557.88	40.12	466.50	646.00	557.04	39.08	0.52^**^	0.68	503.31	602.12	557.46	23.59

Pearson correlation analysis was conducted to examine pairwise correlations among the 12 traits (Fig. 3b). Of these traits, GY was positively correlated with MD, FP, PH, and KPS (*r* = 0.21, 0.21, 0.21, and 0.30, respectively), but negatively correlated with SP and SN (*r* = − 0.25 and − 0.25, respectively). In addition, GY showed a weak positive correlation with TKW and GFR (0.19 for both but not significant). A strong negative correlation was observed between TKW and KPS (*r* = − 0.37). In addition, a negative correlation was observed between SN with TKW (*r* = − 0.44) and KPS (*r* = − 0.34).

For period-related traits, MD and FP were positively correlated with TKW (*r* = 0.25), negatively correlated with SN (*r* = − 0.29), and not significantly correlated with KPS. In addition, both traits showed a positive correlation with HD (*r* = 0.50) but no significant correlation with GFP. On the other hand, HD showed a strong negative correlation with GFP (*r* = − 0.81).

### Phenotypic trait dominance among subpopulations

**Fig. 4:**
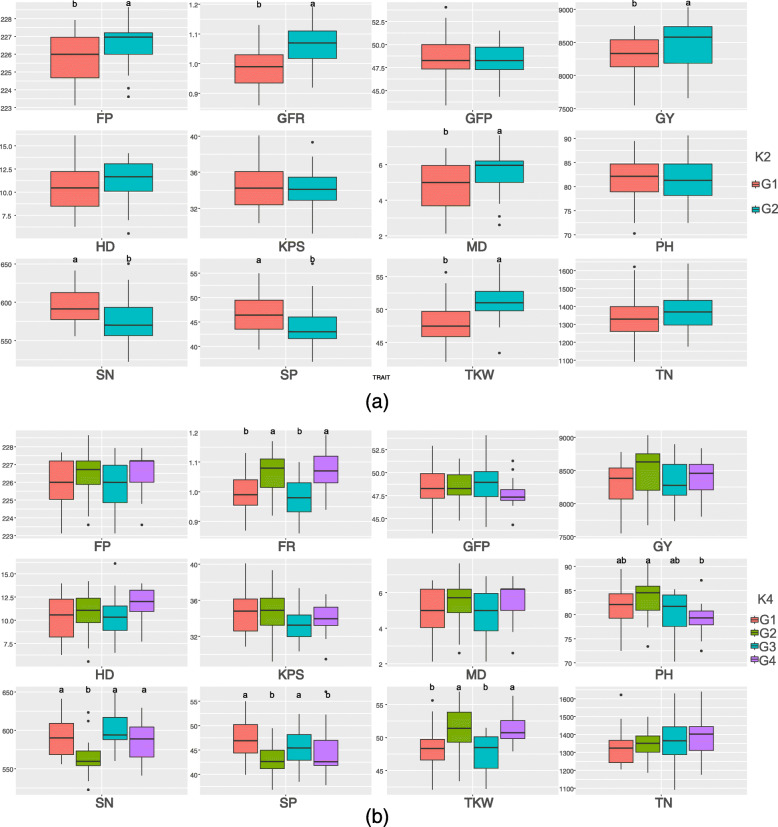
The boxplot of phenotype in different subpopulations when K = 2 **a** and K = 4 **b**. ANOVA was based on scheffe multiple comparisons; the letters above the boxes indicate significant differences between the alleles (*p* < 0.05)

The phenotypic dominance among subpopulations at *K* = 2 and *K* = 4 was assessed, and was more strongly observed at *K* = 2 (Fig. 4a) than under *K* = 4 (Fig. 4b).

Phenotypic dominance was not significant for GFP, HD, KPS, and TN among subpopulations either under *K* = 2 or *K* = 4. The phenotypic dominance for TKW and GFR in subpopulation 2:2 was significantly higher than that in subpopulation 2:1 under *K* = 2, with the reverse trend observed for SP and SN. However, these phenotypic differences were not detected together under *K* = 4. In addition, the phenotypic values of GY, MD, and FP in supopulation 2:2 were significantly higher than those in subpopulation 2:1, but significance was not detected under *K* = 4. In addition, the predominant phenotype for PH was observed in subpopulation 2:2 under *K* = 4, for which the plants grouped in Sp4 were shorter than those in Sp2.

### Genome-wide association study of 12 agronomic traits

A total of 107 significant SNPs (*p* < 0.001) distributed across all 21 chromosomes except 3D, 4A, 4D, and 6D were detected for GY and related traits. With regard to the individual traits, 13, 11, 15, 10, 7, 3, 13, 6, 21, 7, 12, and 6 significant SNPs were detected for FP, GFR, GFP, GY, HD, KPS, MD, PH, SN, SP, TKW, and SN, respectively. These significant loci showed logarithm of the odds scores ranging from 3.00 to 5.74 and contributed 10.45–24.19% of the effects on the corresponding traits (Table S4, Fig. S2).

**Fig. 5:**
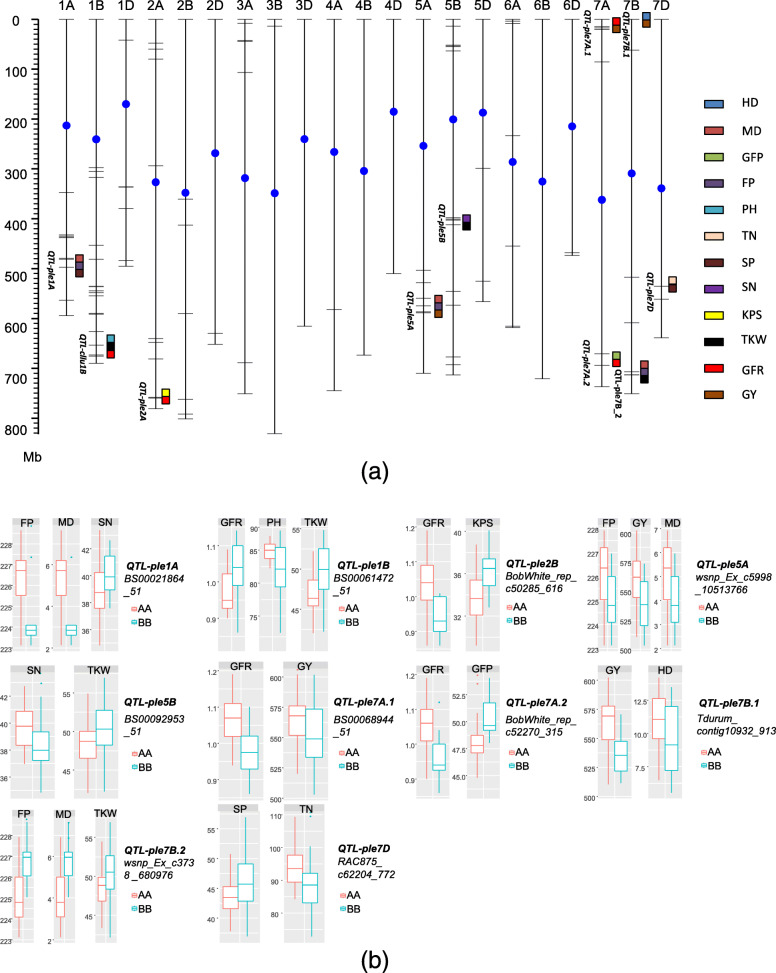
phenotype distribution for biallelic of represented SNPs in 10 pleiotropic QTL regions. **a** Whole genome distribution of ten ploeitropic QTLs. The labels on the left and right of chromosome were the name of pleotropic QTL and underlying traits. The color showed different traits marked on the right of the figure. **b** Haplotype of underlying traits for each of ten pleiotropic QTLs

Ten QTL regions showed pleiotropic contributions to the various traits with consideration of the average LD decay distance. The QTLs were distributed on chromosomes 1A, 1B, 2A, 5A, 5B, 7A (2), 7B (2), and 7D (Fig. 5).

Three QTL regions showed pleiotropic effects on GY with one or two other traits. The QTL *QTL-ple7A.1*, represented by the marker *BS00068944_51*, controlled GY and GFR with consistent effect directions. Effects in the same direction were also observed for HD and GY, and for FP, MD, and GY explained by *QTL-ple7B.1* and *QTL-ple5A*, respectively, which were represented by the SNP markers *Tdurum_contig10932_913* and *wsnp_Ex_c5998_10513766*, respectively.

The traits MD/FP were co-located with other traits in two additional QTL regions: with SN at *QTL-ple1A* (represented by *BS00021864_51*) with opposite effect directions, and co-located with TKW at *QTL-ple7B.2* (represented by *wsnp_Ex_c3738_6809767*) with consistent effect directions.

The GFR was co-located with four other traits within three QTL regions with differing effect directions. The traits KPS and GFP were respectively controlled by *QTL-ple2B* (*BobWhite_rep_c50285_616*) and *QTL-ple7A.2* (*BobWhite_rep_c52270_315*) with GFR with contrasting effect directions. The QTL *QTL-ple1B* (*BS00061472_51*) was co-located with TKW with the same effect direction, but with PH in the opposite effect direction.

The TKW value of the allele *QTL-ple5B_BB* was higher than that for *QTL-ple5B_AA*, whereas the opposite result was observed for SP. In addition, the complementary effect direction was observed for *QTL-ple7D* controlling SP and TN.

### Whole-genome scanning of selective sweep signal

**Fig. 6:**
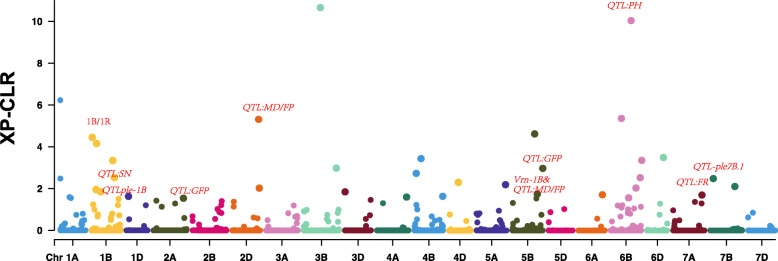
Whole genome scanning of selective sweeps (subpopulation 2:2 against subpopulation 2:1)

To investigate the effects of candidate selective sweeps on divergence of traits across the whole genome, we searched for signatures of selection by comparison of two subpopulations under *K* = 2. The footprints of selection were detected in a total of 62 genomic regions across all 21 chromosomes except 5D and 7D, with a span of 0.83% of the wheat genome. The mean strength of selection was 0.0305 (Table S5, Fig. 6).

Ten selective sweep regions were around QTLs for agraonomic traits. Two selection signals located at 623251480 bp and 637,251,480 bp flanked *QTL-ple1B.* Two signals on chromosomes 2A and 5B were located about ~ 10 Mb from two QTLs for GFP. Similarly, a selective sweep signal on chromosome 1B at 535251480 bp was ~ 18 kb from a QTL for SN. Signals on chromosome 5B at 562087488 bp and 580,087,488 bp flanked QTLs for MD and FP, which covered the *vrn-B1* gene region that was previously reported to be involved in the regulation of growth habit [15]. In addition, five selection signals were detected on chromosome 1BS, which indicated that the 1B/1R translocation was an important signature for population inferior.

## Discussion

### Plant architecture among subpopulations is shaped by ecology

Previous studies of wheat have shown that subpopulation structure is dependent on the geographic origin or the status of domestication [16–18]. In the present study, the population structure of a panel of commercial cultivars from Henan was mainly determined by inheritance of characters from the donor parents, which significantly affected phenotypic variation in relation to breeding targets and environmental adaptation. The 1B/1R translocation and external genetic resources were additional factors that also impacted on population structure.

The temperature in Henan generally decreases from southern to northern latitudes, but the ecological factors in different regions provide different selection pressures and have shaped plant architecture and growing season. The northwestern region of Henan experiences a longer winter but with higher temperatures because the Tai-hang Mountains block penetration of cold air from Siberia, which is the reason that cultivars grouped in subpopulation 2:1 selected in this region inherited early maturity from Yanshi 4 and strong tillering ability from Yumai 2. By contrast, the centre-eastern region of Henan is a flat plain that is vulnerable to the influence of the winter monsoonal climate of moderate latitudes. Cultivars grouped in subpopulation 2:2 are suited for cultivation on the southeastern plain and were selected for desirable grain filling characters derived from Zhou 8425B [19]. Further subdivision of subpopulations from *K* = 2 to *K* = 4 only slightly influenced phenotypic differentiation, which indicated that introduction of cultivars from other regions probably contributed to other traits, such as end-use quality and cold resistance.

### Genetic basis of yield and its improvement

Improvement of yield has been an important objective in the breeding of modern wheat cultivars. Yield comprises three major traits: kernel weight, kernel number per spike, and spike number. However, each of these three components contribute to yield improvement to different degrees [20–22]. In the present study, the three yield components were strongly coordinated. Grain yield was positively correlated with KPS, negatively correlated with SN, and weakly positively correlated with TKW, which indicated that improvement of yield was dependent on increased sink capacity of the panicle versus vegetative reproduction.

The determination of grain yield is complex and factors at any stage of the growth cycle may influence final yield in continental wheat production areas [23]. In the current study, GY was positively correlated with FP, MD, and HD, which implied that a long reproductive growth period is an important factor in source supply [24, 25]. In contrast, a shortened FP is beneficial to increase the cropping index (a maize–wheat cropping system is commonly practiced in Henan), which demonstrates that improvement of yield by accelerating the growth rate is a challenge. However, no correlation was observed between MD/FP and KPS, which suggested that increase in KPS for yield improvement is less sensitive than TKW to MD/FP.

Previous studies have demonstrated that GY enhancement is positively correlated with reduced plant height by pyramiding semi-dwarf genes that drastically decrease PH from more than 100 cm to ~ 80 cm [26–31]. The positive correlation between PH and GY in the current study was possibly because low plant height would lead to loss of biomass and increase in disease risk, and plant height is not the main cause of lodging in wheat [32–37].

Identification of pleiotropic QTLs in an association-mapping population is a promising method to dissect effectively the genetic basis of related traits [7, 38–42]. Identification of QTLs that control the trade-off among spike traits has been widely reported [10, 43]. The QTL *QTL-ple2B* showed pleiotropic effects on GFR and KPS in the present study. A recently cloned gene, *GNI*, on chromosome 2A shows pleiotropic effects on KPS and TKW [44]. However, owing to the location of its homoeologues on 2B, *QTL-ple2B* is not the same gene [44, 45]. Given the high correlation between GFR and TKW, *QTL-ple2B* is a novel locus that explains the genetic basis of the trade-off between TKW (GFR) and SN. In addition, *QTL-ple5B* complementarily regulated TKW and SN, which are two negatively correlated traits. This QTL interval seems to be a novel allele for kernel-related traits. However, a recently reported stable QTL on chromosome 5A associated with 6.9% increase in grain weight [46]. This QTL interval is probably located in the collinearity region with *QTL-ple5B* but needs to be further explored. An additional QTL, *QTLple-7D*, controlled both TN and SP in contrasting directions, which suggested that the promotion of tiller formation during early development could not be entirely transformed into effective spikes. Furthermore, *QTLple-7D* was a novel QTL because no QTL has been documented to control tiller development.

The alleles *QTL-ple5A*, *QTL-ple7B.2*, and *QTL-ple1A* that contribute to longer FP were associated with higher GY, higher TKW, and lower SN, respectively, which provides a genetic basis for the effect of maturity period on grain yield and yield components. In particular, *QTL-ple5A* and *QTL-ple7B.2* were located in similar genomic positions to *VRN-1A* and *VRN-3A* according to the Chinese Spring reference assembly genome, which is consistent with several previous reports on diverse panels of association-mapping populations indicating that *vrn1* and *vrn3* are associated with GY [38, 47–50]. In addition, the gene underlying *QTL-ple1A* has not been identified, which suggested that this locus represented a novel gene.

### Selective QTLs

Extensive studies of plants have detected population genetic signatures around significantly associated loci, with various divisions of subpopulations, such as domesticated versus wild accessions [12, 51], and that plant architecture is plastic in response to ecological variation [13] or subspecies divergence [52].

Several selective sweep signals flanking known functional genes were identified in the present study, including the previously reported adaptation gene *VRN-1B*, which exhibits dominance effects to *VRN-1A* on grain yield [49, 53]. In this study, prolonging MD/FP by selective allele *VRN-1B* have non-significant effects on GY. In contrast, *QTLple-5A* (*VRN-1A*) extending MD/FP could also enhance GY These results suggested that a breeding strategy to explore the balance between early maturity and high yield may be possible by pyramiding *VRN-1B* only.

Several selective sweep regions that flanked QTLs for agronomic traits (e.g., TN, GFR, MD, and FP) were identified in the present study, which reflected that these alleles were contributed by utilization of characters of the donor parents, possibly in response to ecological environments. In particular, no selective sweep signals were detected around QTLs for KPS, which suggested that alleles contributing to increase in KPS may be further applicable in breeding.

## Conclusions

Genome-wide association analysis identified several QTLs associated with grain yield and yield components, and provided insight into the genetic basis of yield-related traits. Some of the identified QTLs were under selection, which implied that extensive changes in allele frequency were driven by ecological pressure to shape plant architecture and growing seasons. We explored the relationships among population structure, ecological adaptation, QTLs for agronomic traits, and selective regions. The identified QTLs *QTLple-7A.1* and *QTLple-5A* may be useful for yield improvement in wheat by accelerating grain filling and moderately delaying maturity date, respectively. Furthermore, the QTLs not under selective pressure can be used for marker-assisted selection in breeding for trait improvement.

## Method

### Plant materials and field trials

A panel of 96 wheat cultivars (Table S1) from the South of Yellow and Huai Valleys of China were selected for the study [54, 55]. These cultivars were cultivated across the northwest to centre-east regions of Henan province and saved in Institute of Wheat, Henan Academy of Agraicultural Sciences. The cultivars were grown at two locations (Xinxiang and Anyang) using a randomized block experimental design and the seeds were sown mechanically. For all experiments, two replicate plots each of 8 m × 1.2 m, with 150,000 seeds per plot, were established for each cultivar.

### Genotyping and physical mapping

The genomic DNA of each cultivar was extracted from young leaf tissues using the cetyl trimethylammonium bromide method and were genotyped using the Illumina iSelect® 90 K SNP Assay, which was performed at the University of California at Davis Genome Center (Davis, CA, USA). A local library derived from the wheat Chinese Spring reference genome sequence (IWGSC v1.0; https://wheat-urgi.versailles.inra.fr/) was constructed using the BLAST+ 2.2.25 package (National Center for Biotechnology Information, Bethesda, MD, USA) to search for the top hits of all sequences flanking the single-nucleotide polymorphism (SNP) markers to determine their physical positions (Table S2).

The genotypic clusters for each SNP were determined using Genome Studio version 2011.1 software (Illumina, https: //www.illumina.com). The genotypes based on 81,587 probes for all samples were classified into two homozygous (AA&BB) and one heterozygous (AB) corresponding to the genotypes expected for biallelic SNPs. The SNPs with missing rate > 5% were removed and 80,540 SNPs were retained.

### Population analysis

Population structure was estimated using a model-based approach implemented in STRUCTURE version 2.3 software under different numbers of clusters (*K*) ranging from *K* = 1 to *K* = 10. An admixture model with 10,000 burn-in iterations, followed by 10,000 Markov chain Monte Carlo iterations, for accurate parameter estimation was used with five independent replicates for each *K* value [56]. The STRUCTURE HARVESTER online program (http://taylor0.biology.ucla.edu/structureHarvester/) was used to collate the STRUCTURE output files to detect the most likely level of population subdivision using the Evanno method [57]. Principal component analysis (PCA) was conducted using GAPIT with the default parameters to calculate the first three principal components (PCs) [58]. A neighbour-joining tree was constructed using MEGA version 7.0.14 using the Kimura 2-parameter model [59]. Bootstrap tests were performed with 1000 replications to assess statistical support for the tree topology. The final tree was visualized using the “ggtree” package [60] using R scripts.

Linkage disequilibrium (LD) analysis was performed using the square of the correlation coefficient (*r*^2^) for each pair of markers on each chromosome using TASSEL version 5.2.55 [61, 62]. The computation used a sliding window of 50 markers around the current site and a cutoff of *p* < 0.001. The mean *r*^2^ in a 0.5 Mb window was plotted against physical distance and an exponential curve was fitted to the data. The corresponding physical distance at which *r*^2^ decayed to 50% of the maximum was determined to be the average decay distance.

The genetic parameters number of variable sites, nucleotide diversity (π), average number of nucleotide differences, and number of haplotypes across subgroups and overall were calculated using DnaSP version 6.12.03 [63].

### Phenotype assessment and analysis

Twelve agronomic traits were recorded, comprising heading date (HD), maturity date (MD), grain filling period (GFP), fertility period (FP), tiller number (TN), spike percentage (SP), spike number (SN), plant height (PH), number of kernels per spike (KPS), thousand-kernel weight (TKW), grain filling rate (GFR), and grain yield (GY).

The sowing date was identical (7 October) at both locations. The HD and MD dates were determined as 50% of spike heading and maturity for whole plots, respectively. The GFP was calculated as MD minus HD; FP was equal to MD owing to the common sowing date. The MD and HD were recorded as relative value by compared the real and the earliest value. For evaluation of TN, KPS, PH, SN, and TKW, a sample row of 1 m length was monitored from the seedling stage until harvest. The TN was calculated as the total number of tillers before jointing; SN was measured as the total number of spikes. The values for TN and SP were converted to total number per plot. The PH was measured as the distance between the top of the spike and the base of the root; KPS and TKW were calculated as follows:

$$ \mathrm{KPS}=\frac{\mathrm{number}\ \mathrm{of}\ \mathrm{total}\ \mathrm{seeds}\ \mathrm{harvested}\ \mathrm{in}\ \mathrm{sample}}{\mathrm{total}\ \mathrm{spike}\ \mathrm{number}} $$

$$ \mathrm{TKW}=\frac{\mathrm{total}\ \mathrm{weight}\ \mathrm{of}\ \mathrm{sampled}\ \mathrm{seeds}\ }{\mathrm{number}\ \mathrm{of}\ \mathrm{total}\ \mathrm{seeds}}\ast 1000 $$

The GFR was determined as TKW divided by GFP; GY was calculated as the total grain weight per plot.

The best linear unbiased prediction (BLUP) for each trait in the two locations was inferred using the “lme4” package for R and fitted using a linear mixed model [64]:

$$ Y=G+L+G\ast L+G\ast L+r\%L+e $$

where ***Y*** is the phenotype, ***G*** is the genotyping effect, ***L*** is the location effect, ***r*** is the number of replicates, * represents interaction between two factors, % represents the replication nest in location factor, and ***e*** is the residual.

The broad-sense heritability (*H*^2^) was estimated using the following equation:

$$ {H}^2=\frac{\sigma_G^2}{\sigma_G^2+\frac{\sigma_{G\ast L}^2}{\overline{L}}+\frac{\sigma_e^2}{\overline{rL}}} $$

where ${\sigma}_G^2$ is the variance component of genotypes, ${\sigma}_{G\ast L}^2$ is the variance component of the interaction between genotype and location, ${\sigma}_e^2$ is the variance of the residual, *r* is the number of replicates at each location, and *L* is the harmonic mean of year and location per tested cultivar [65].

Calculation of pairwise correlation coefficients for these traits was conducted with the *cor* function using the “pearson” method at the 5% significance level (*p* < 0.05) and was visualized using the “corrplot” package in R [66].

To address phenotype dominance among four subgroups individually, one-way analysis of variance (ANOVA) was used based on the Scheffe multiple comparison method at the 5% significance level (*p* < 0.05) using IBM SPSS Statistics version 22 (IBM Corporation, Armonk, NY, USA).

### Association mapping

Association mapping was conducted using a mixed linear model with TASSEL version 5.0 [62]. In total, 11,930 SNPs of known physical position and less than 20% missing values were used, and all heterozygous genotypes were excluded from the marker-traits association. Population structure (*Q* matrix), which was defined as the differential relatedness among genotypes, and the kinship matrix (*K* matrix) representing the proportion of shared alleles for all pairwise comparisons in each population were included in the statistic. Genome wide association mapping was conducted using MLM model (mixed linear model) as describe in TASSEL [62]. The BLUP values for each trait were used for phenotypic observation. The threshold −log10 (*P*-value) ≥ 3.0 for each marker was regarded as significant for genome-wide association mapping.

Given the systematic error of phenotype measurement, screening of pleiotropic QTLs was conducted using the following criteria: 1) markers for different traits within the average LD distance were first regarded as one QTL; and 2) all cultivars were grouped by each biallelic SNP and the significance of the phenotype was tested by ANOVA (*p* < 0.05), for which the aim was to select one marker that represented the QTL in the following haplotype analysis. Alternatively, all significant markers within the average LD that did not pass the ANOVA for any traits would be excluded for pleiotropic QTL screening.

For the subsequent haplotype analysis, a subset of phenotype parameters corresponding to each homozygous allele of a representive marker was analyzed to reveal the QTL effects on the traits (using Scheffe’s method, *p* < 0.05).

### Selective sweep analysis

A cross-population composite likelihood ratio test, implemented in XP-CLR version 1.0 [67], was used to scan selective sweeps in the comparison between subgroups under *K* = 2 representing trait dominance. The SNPs with neither more than 80% deletion nor minor allele frequency less than 5% were excluded from the analysis. Genetic distances between adjacent SNPs were calculated on the basis of the proportionally increased physical distance of adjacent surrounding markers in an integrated genetic map. For each chromosome, the XP-CLR score was calculated with the following command: ‘-w1 0.5 200 $blockSize 1 -p0 0.95’. Regions with region-wise XP-CLR scores in the top 1% were considered to be candidate selective sweeps.

## Supplementary information

**Additional file 1: Figure S1.** (a) Plot of the PCA analysis (PC1&PC3, PC2&PC3) illustrated four subpopulations which assigned in STRUCTURE result. (b) Genome-wide LD (*r*^2^) distribution against the genetic distance. The dotted line marked each 0.2 per *r*^2^.

**Additional file 2: Figure S2.** Genome-wide association studies and quantile-quantile plot of 12 agronomic traits. The trait name was marked in the upper or bottom of each figure. For Manhattan plots, Negative -log10 *P* values from a genome-wide scan are plotted against physical position on each of 21 chromosomes. Red line indicates the genome-wide significance threshold.

**Additional file 3: Table S1.** Information of 96 Henan wheat varieties, including pedigrees and their subpopulation assignment identified in STRUCTURE.

**Additional file 4: Table S2** Information of SNPs and genotypic data for 96 wheat varieties. The chromosome and position information were referred to the physical position in Chinese Spring reference genome (CS Ref v1.0, IWGSC).

**Additional file 5: Table S3.** Comparison of the population genetic parameters in four subpopulations.

**Additional file 6: Table S4.** Information of marker-trait associations, pleiotropic QTLs and candidate genes for 12 agronomic traits.

**Additional file 7: Table S5.** Whole geneome scanning of selective sweep signals for subpopulations at K = 2.

## Data Availability

The phenotypic data of the current study are available upon request through email to the corresponding author. The genotyping data are available in the Additional file 4.

## Abbreviations

QTL: Quantitative trait locus
GWAS: Genome-wide association studies
LD: Linkage disequilibrium
SNP: Single nucleotide polymorphism
BLUP: Best linear unbiased prediction
PCA: Principal component analysis
HD: Heading date
MD: Maturity date
GFP: Grain filling period
FP: Fertility period
TN: Tiller number
SP: Spike percentage
SN: Spike number
PH: Plant height
KPS: Number of kernels per spike
TKW: Thousand kernel weight
GFR: Grain filling rate
GY: Grain yield

## References

[1] Karlsson EK, Kwiatkowski DP, Sabeti PC (2014). Natural selection and infectious disease in human populations. Nat Rev Genet.

[2] Atanur Santosh S, Diaz Ana G, Maratou K, Sarkis A, Rotival M, Game L, Tschannen Michael R, Kaisaki Pamela J, Otto Georg W, man M, Chun J (2013). Genome sequencing reveals loci under artificial selection that underlie disease phenotypes in the laboratory rat. Cell.

[3] Liedvogel M, Åkesson S, Bensch S (2011). The genetics of migration on the move. Trends Ecol Evol.

[4] Zhao K, Tung CW, Eizenga GC, Wright MH, Ali ML, Price AH, Norton GJ, Islam MR, Reynolds A, Mezey J (2011). Genome-wide association mapping reveals a rich genetic architecture of complex traits in Oryza sativa. Nat Commun.

[5] Huang X, Zhao Y, Wei X, Li C, Wang A, Zhao Q, Li W, Guo Y, Deng L, Zhu C (2011). Genome-wide association study of flowering time and grain yield traits in a worldwide collection of rice germplasm. Nat Genet.

[6] Guo Z, Chen D, Alqudah AM, Röder MS, Ganal MW, Schnurbusch T (2017). Genome-wide association analyses of 54 traits identified multiple loci for the determination of floret fertility in wheat. New Phytol.

[7] Li F, Wen W, Liu J, Zhang Y, Cao S, He Z, Rasheed A, Jin H, Zhang C, Yan J (2019). Genetic architecture of grain yield in bread wheat based on genome-wide association studies. BMC Plant Biol.

[8] Quintero A, Molero G, Reynolds MP, Calderini DF (2018). Trade-off between grain weight and grain number in wheat depends on GxE interaction: a case study of an elite CIMMYT panel (CIMCOG). Eur J Agron.

[9] Sadras VO (2007). Evolutionary aspects of the trade-off between seed size and number in crops. Field Crops Res.

[10] Sukumaran S, Lopes M, Dreisigacker S, Reynolds M (2018). Genetic analysis of multi-environmental spring wheat trials identifies genomic regions for locus-specific trade-offs for grain weight and grain number. Theor Appl Genet.

[11] Lu K, Wei L, Li X, Wang Y, Wu J, Liu M, Zhang C, Chen Z, Xiao Z, Jian H (2019). Whole-genome resequencing reveals Brassica napus origin and genetic loci involved in its improvement. Nat Commun.

[12] Guo S, Zhao S, Sun H, Wang X, Wu S, Lin T, Ren Y, Gao L, Deng Y, Zhang J (2019). Resequencing of 414 cultivated and wild watermelon accessions identifies selection for fruit quality traits. Nat Genet.

[13] Wu D, Liang Z, Yan T, Xu Y, Xuan L, Tang J, Zhou G, Lohwasser U, Hua S, Wang H (2019). Whole-genome Resequencing of a worldwide collection of rapeseed accessions reveals the genetic basis of ecotype divergence. Mol Plant.

[14] Cavanagh CR, Chao S, Wang S, Huang BE, Stephen S, Kiani S, Forrest K, Saintenac C, Brown-Guedira GL, Akhunova A (2013). Genome-wide comparative diversity uncovers multiple targets of selection for improvement in hexaploid wheat landraces and cultivars. Proc Natl Acad Sci.

[15] Fu D, Szűcs P, Yan L, Helguera M, Skinner JS, Von Zitzewitz J, Hayes PM, Dubcovsky J (2005). Large deletions within the first intron in VRN-1 are associated with spring growth habit in barley and wheat. Mol Gen Genomics.

[16] Zhou Y, Chen Z, Cheng M, Chen J, Zhu T, Wang R, Liu Y, Qi P, Chen G, Jiang Q (2018). Uncovering the dispersion history, adaptive evolution and selection of wheat in China. Plant Biotechnol J.

[17] Alemu A, Feyissa T, Letta T, Abeyo B (2020). Genetic diversity and population structure analysis based on the high density SNP markers in Ethiopian durum wheat (*Triticum turgidum ssp. durum*). BMC Genet.

[18] Balfourier F, Bouchet S, Robert S, De Oliveira R, Rimbert H, Kitt J, Choulet F, Paux E, Consortium IWGS, Consortium B: Worldwide phylogeography and history of wheat genetic diversity. Sci Adv 2019, 5(5):eaav0536. 10.1126/sciadv.aav0536 PMC6541461 31149630

[19] Jiao J, Kang W. Analysis on regional characters of the annual rainfall in Henan Province [J]. J North China Institut Water Conserv Hydroelectric Power. 2007:4.

[20] Sidwell RJ, Smith EL, McNew RW (1976). Inheritance and Interrelationships of Grain Yield and Selected Yield-Related Traits in a Hard Red Winter Wheat Cross1. Crop Sci.

[21] Mohammadi M, Sharifi P, Karimizadeh R, Shefazadeh MK (2012). Relationships between grain yield and yield components in bread wheat under different water availability (dryland and supplemental irrigation conditions). Notulae Botan Horti Agrob Cluj-Napoca.

[22] Ferrante A, Cartelle J, Savin R, Slafer GA (2017). Yield determination, interplay between major components and yield stability in a traditional and a contemporary wheat across a wide range of environments. Field Crops Res.

[23] Slafer GA (1996). Differences in phasic development rate amongst wheat cultivars independent of responses to photoperiod and vernalization. A viewpoint of the intrinsic earliness hypothesis. J Agric Sci.

[24] Reynolds M, Balota M, Delgado M, Amani I, Fischer R (1994). Physiological and morphological traits associated with spring wheat yield under hot**, Irrigated Conditions**. Funct Plant Biol.

[25] Shearman VJ, Sylvester-Bradley R, Scott RK, Foulkes MJ (2005). Physiological processes associated with wheat yield Progress in the UK. Crop Sci.

[26] Peng J, Richards DE, Hartley NM, Murphy GP, Devos KM, Flintham JE, Beales J, Fish LJ, Worland AJ, Pelica F (1999). ‘Green revolution’genes encode mutant gibberellin response modulators. Nature.

[27] Bellucci A, Torp AM, Bruun S, Magid J, Andersen SB, Rasmussen SK. Association Mapping in Scandinavian Winter Wheat for Yield, Plant Height, and Traits Important for Second-Generation Bioethanol Production. Front Plant Sci. 2015;6(1046). 10.3389/fpls.2015.01046 PMC4660856 26635859

[28] Beche E, Benin G, da Silva CL, Munaro LB, Marchese JA (2014). Genetic gain in yield and changes associated with physiological traits in Brazilian wheat during the 20th century. Eur J Agron.

[29] Rebetzke G, Ellis M, Bonnett D, Condon A, Falk D, Richards R (2011). The Rht13 dwarfing gene reduces peduncle length and plant height to increase grain number and yield of wheat. Field Crop Res.

[30] Gale MD, Youssefian S, Russell G (1985). Dwarfing genes in wheat. Prog Plant Breed.

[31] Flintham J, Börner A, Worland A, Gale M (1997). Optimizing wheat grain yield: effects of Rht (gibberellin-insensitive) dwarfing genes. JAS.

[32] Miralles D, Slafer G (1995). Yield, biomass and yield components in dwarf, semi-dwarf and tall isogenic lines of spring wheat under recommended and late sowing dates. Plant Breed.

[33] Gent MP (1995). Canopy light interception, gas exchange, and biomass in reduced height isolines of winter wheat. Crop Sci.

[34] Zhang X, Yang S, Zhou Y, He Z, Xia X (2006). Distribution of the Rht-B1b, Rht-D1b and Rht8 reduced height genes in autumn-sown Chinese wheats detected by molecular markers. Euphytica.

[35] Lopes MS, Reynolds MP, Manes Y, Singh RP, Crossa J, Braun HJ (2012). Genetic yield gains and changes in associated traits of CIMMYT spring bread wheat in a “historic” set representing 30 years of breeding. Crop Sci.

[36] Ma D-q, Wang X-w, Xu L-j, Zhu Y-p, K-h Z, Wang D-m. Distribution of dwarfing genes derived from some wheat germplasms in Huang-huai wheat area. J Henan Agricult Univ. 2009;2.

[37] Mesterhazy A (1995). Types and components of resistance to Fusarium head blight of wheat. Plant Breed.

[38] Kato K, Miura H, Sawada S (2000). Mapping QTLs controlling grain yield and its components on chromosome 5A of wheat. Theor Appl Genet.

[39] Gao F, Wen W, Liu J, Rasheed A, Yin G, Xia X, Wu X, He Z (2015). Genome-wide linkage mapping of QTL for yield components, plant height and yield-related physiological traits in the Chinese wheat cross Zhou 8425B/Chinese spring. Front Plant Sci.

[40] Kumar N, Kulwal PL, Balyan HS, Gupta PK (2006). QTL mapping for yield and yield contributing traits in two mapping populations of bread wheat. Mol Breeding.

[41] Li S, Jia J, Wei X, Zhang X, Li L, Chen H, Fan Y, Sun H, Zhao X, Lei T (2007). A intervarietal genetic map and QTL analysis for yield traits in wheat. Mol Breeding.

[42] Korte A, Farlow A (2013). The advantages and limitations of trait analysis with GWAS: a review. Plant Methods.

[43] Zhai H, Feng Z, Du X, Song Y, Liu X, Qi Z, Song L, Li J, Li L, Peng H (2018). A novel allele of TaGW2-A1 is located in a finely mapped QTL that increases grain weight but decreases grain number in wheat (Triticum aestivum L.). Theor Appl Genet.

[44] Sakuma S, Golan G, Guo Z, Ogawa T, Tagiri A, Sugimoto K, Bernhardt N, Brassac J, Mascher M, Hensel G (2019). Unleashing floret fertility in wheat through the mutation of a homeobox gene. Proc Natl Acad Sci U S A.

[45] Golan G, Ayalon I, Perry A, Zimran G, Ade-Ajayi T, Mosquna A, Distelfeld A, Peleg Z (2019). GNI-A1 mediates trade-off between grain number and grain weight in tetraploid wheat. Theor Appl Genet.

[46] Brinton J, Simmonds J, Minter F, Leverington-Waite M, Snape J, Uauy C (2017). Increased pericarp cell length underlies a major quantitative trait locus for grain weight in hexaploid wheat. New Phytol.

[47] Stelmakh AF (1992). Genetic effects of Vrn genes on heading date and agronomic traits in bread wheat. Euphytica.

[48] Zhang LY, Liu DC, Guo XL, Yang WL, Sun JZ, Wang DW, Zhang A (2010). Genomic distribution of quantitative trait loci for yield and yield-related traits in common wheat. J Integr Plant Biol.

[49] Ogbonnaya FC, Rasheed A, Okechukwu EC, Jighly A, Makdis F, Wuletaw T, Hagras A, Uguru MI, Agbo CU (2017). Genome-wide association study for agronomic and physiological traits in spring wheat evaluated in a range of heat prone environments. Theor Appl Genet.

[50] Bennett D, Reynolds M, Mullan D, Izanloo A, Kuchel H, Langridge P, Schnurbusch T (2012). Detection of two major grain yield QTL in bread wheat (Triticum aestivum L.) under heat, drought and high yield potential environments. Theor Appl Genet.

[51] Hufford MB, Xu X, van Heerwaarden J, Pyhajarvi T, Chia JM, Cartwright RA, Elshire RJ, Glaubitz JC, Guill KE, Kaeppler SM (2012). Comparative population genomics of maize domestication and improvement. Nat Genet.

[52] Du X, Huang G, He S, Yang Z, Sun G, Ma X, Li N, Zhang X, Sun J, Liu M (2018). Resequencing of 243 diploid cotton accessions based on an updated a genome identifies the genetic basis of key agronomic traits. Nat Genet.

[53] Sehgal D, Autrique E, Singh R, Ellis M, Singh S, Dreisigacker S (2017). Identification of genomic regions for grain yield and yield stability and their epistatic interactions. Sci Rep.

[54] Yang J, Zhou Y, Wu Q, Chen Y, Zhang P, Zhang Y, Hu W, Wang X, Zhao H, Dong L (2019). Molecular characterization of a novel TaGL3-5A allele and its association with grain length in wheat (Triticum aestivum L.). Theor Appl Genet.

[55] Yang J, Zhou Y, Zhang Ye HW, Wu Q, Chen Y, Wang X, Guo G, Liu Z, Cao T, et al. Cloning, characterization of TaGS3 and identification of allelic variation associated with kernel traits in wheat (*Triticum aestivum* L.). BMC Genet. 2019;20(1). 10.1186/s12863-019-0800-6 PMC6921503 31852431

[56] EVANNO G, REGNAUT S, GOUDET J (2005). Detecting the number of clusters of individuals using the software structure: a simulation study. Mol Ecol.

[57] Earl DA, vonHoldt BM (2012). STRUCTURE HARVESTER: a website and program for visualizing STRUCTURE output and implementing the Evanno method. Conserv Genet Resour.

[58] Lipka AE, Tian F, Wang Q, Peiffer J, Li M, Bradbury PJ, Gore MA, Buckler ES, Zhang Z (2012). GAPIT: genome association and prediction integrated tool. Bioinformatics.

[59] Kimura M: A simple method for estimating evolutionary rate of base substitutions through comparative studies of nucleotide sequences (1980) Proc. In: *Natl Acad Sci USA*. 1181–1188. 10.1007/BF01731581 7463489

[60] Yu G, Smith DK, Zhu H, Guan Y, TTY L (2017). Ggtree: an R package for visualization and annotation of phylogenetic trees with their covariates and other associated data. Methods Ecol Evol.

[61] Weir BS, Ott J (1997). Genetic data analysis II. Trends Genet.

[62] Bradbury PJ, Zhang Z, Kroon DE, Casstevens TM, Ramdoss Y, Buckler ES (2007). TASSEL: software for association mapping of complex traits in diverse samples. Bioinformatics.

[63] Rozas J, Ferrer-Mata A, Sánchez-DelBarrio JC, Guirao-Rico S, Librado P, Ramos-Onsins SE, Sánchez-Gracia A (2017). DnaSP 6: DNA sequence polymorphism analysis of large data sets. Mol Biol Evol.

[64] Bates D, Mächler M, Bolker B, Walker S: **Fitting linear mixed-effects models using lme4**. *arXiv preprint arXiv:14065823* 2014.

[65] Piepho H-P, Möhring J (2007). Computing heritability and selection response from unbalanced plant breeding trials. Genetics.

[66] Wei T, Simko V, Levy M, Xie Y, Jin Y, Zemla J (2017). Package ‘corrplot’. Statistician.

[67] Chen H, Patterson N, Reich D (2010). Population differentiation as a test for selective sweeps. Genome Res.

