# Supplementary figures and images for: Unlocking the relationships among population structure, plant architecture, growing season, and environmental adaptation in Henan wheat cultivars
Source: BMC Plant Biol. 2020 Oct 12;20:469. doi: 10.1186/s12870-020-02674-z (PMC7552505; doi:10.1186/s12870-020-02674-z)

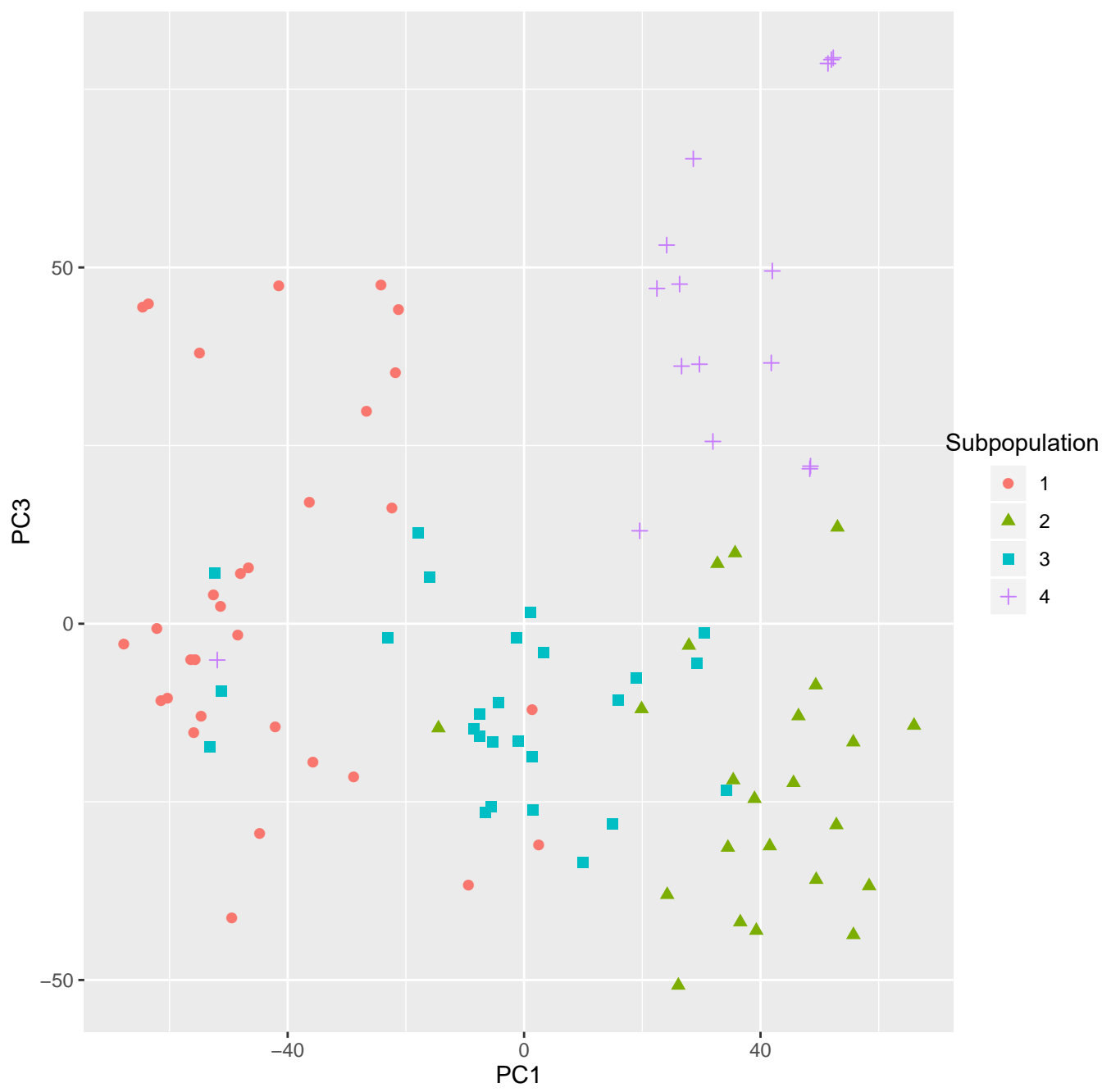

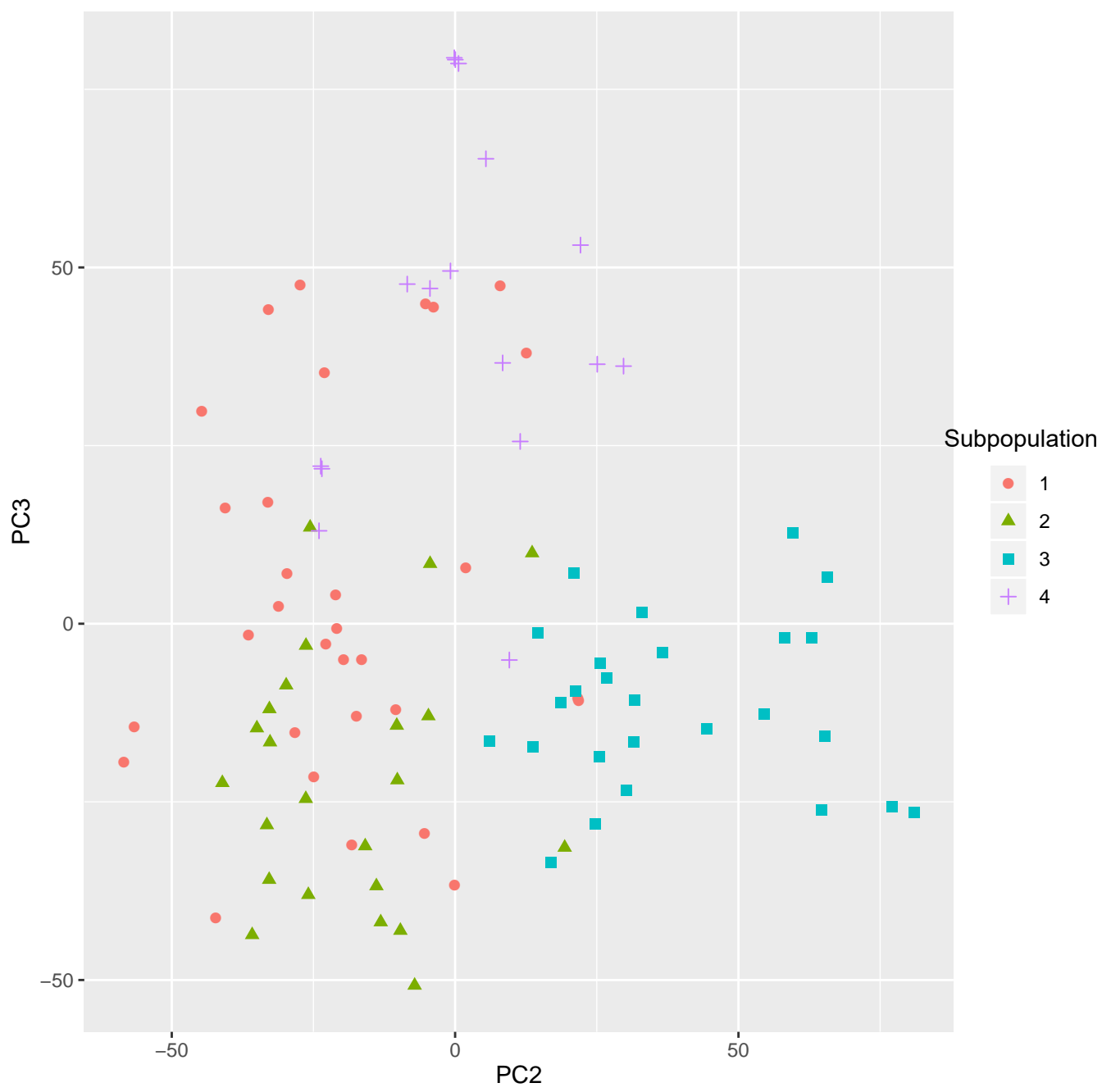

Supplement: Supplementary file 1 — Additional file 1: Figure S1. (a) Plot of the PCA analysis (PC1&PC3, PC2&PC3) illustrated four subpopulations which assigned in STRUCTURE result. (b) Genome-wide LD (r2) distribution against the genetic distance. The dotted line marked each 0.2 per r2. [file 12870_2020_2674_MOESM1_ESM.zip › Additional file 1 Figure S1a PCA result PC1&PC2&PC3.pdf]

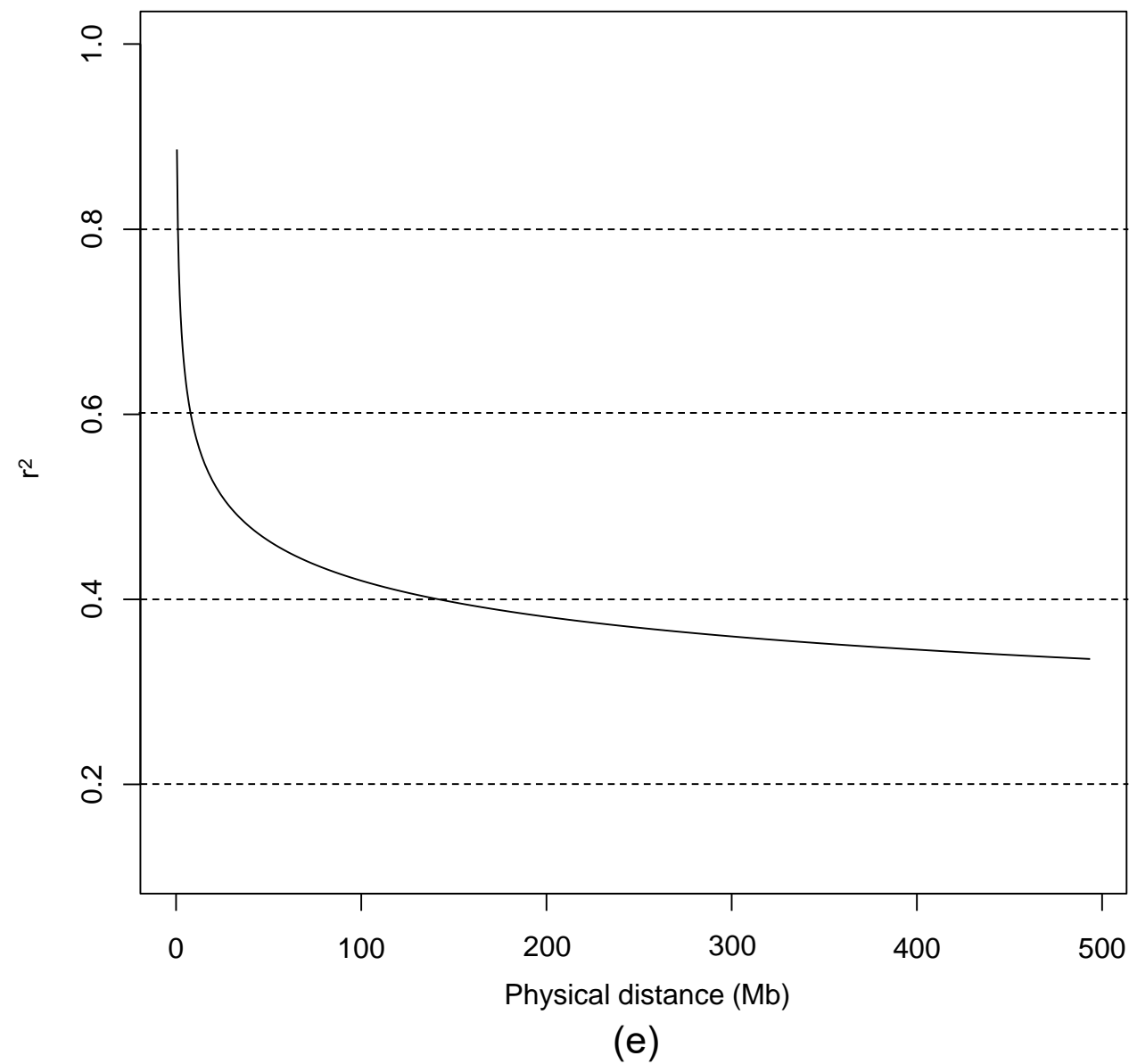

Supplement: Supplementary file 1 — Additional file 1: Figure S1. (a) Plot of the PCA analysis (PC1&PC3, PC2&PC3) illustrated four subpopulations which assigned in STRUCTURE result. (b) Genome-wide LD (r2) distribution against the genetic distance. The dotted line marked each 0.2 per r2. [file 12870_2020_2674_MOESM1_ESM.zip › Additional file 1 Figure S1b LD.pdf]
