# Supplementary material for: Unlocking the relationships among population structure, plant architecture, growing season, and environmental adaptation in Henan wheat cultivars
Source: BMC Plant Biol. 2020 Oct 12;20:469. doi: 10.1186/s12870-020-02674-z (PMC7552505; doi:10.1186/s12870-020-02674-z)
Supplement: Supplementary file 2 — Additional file 2: Figure S2. Genome-wide association studies and quantile-quantile plot of 12 agronomic traits. The trait name was marked in the upper or bottom of each figure. For Manhattan plots, Negative -log10 P values from a genome-wide scan are plotted against physical position on each of 21 chromosomes. Red line indicates the genome-wide significance threshold. [file 12870_2020_2674_MOESM2_ESM.pdf]

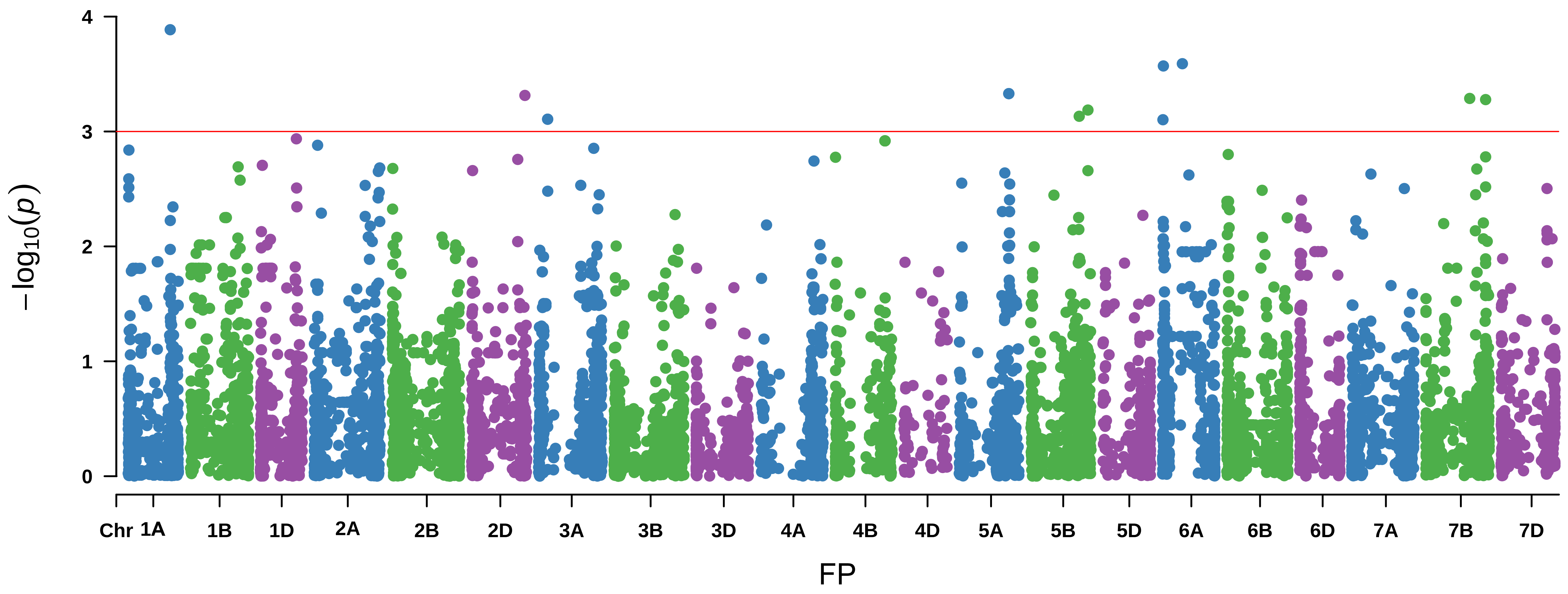

4
3
2
1
0
−log10(p)
Chr 1A
1B
1D
2A
2B
2D
3A
3B
3D
4A
4B
4D
5A
5B
5D
6A
6B
6D
7A
7B
7D
FP

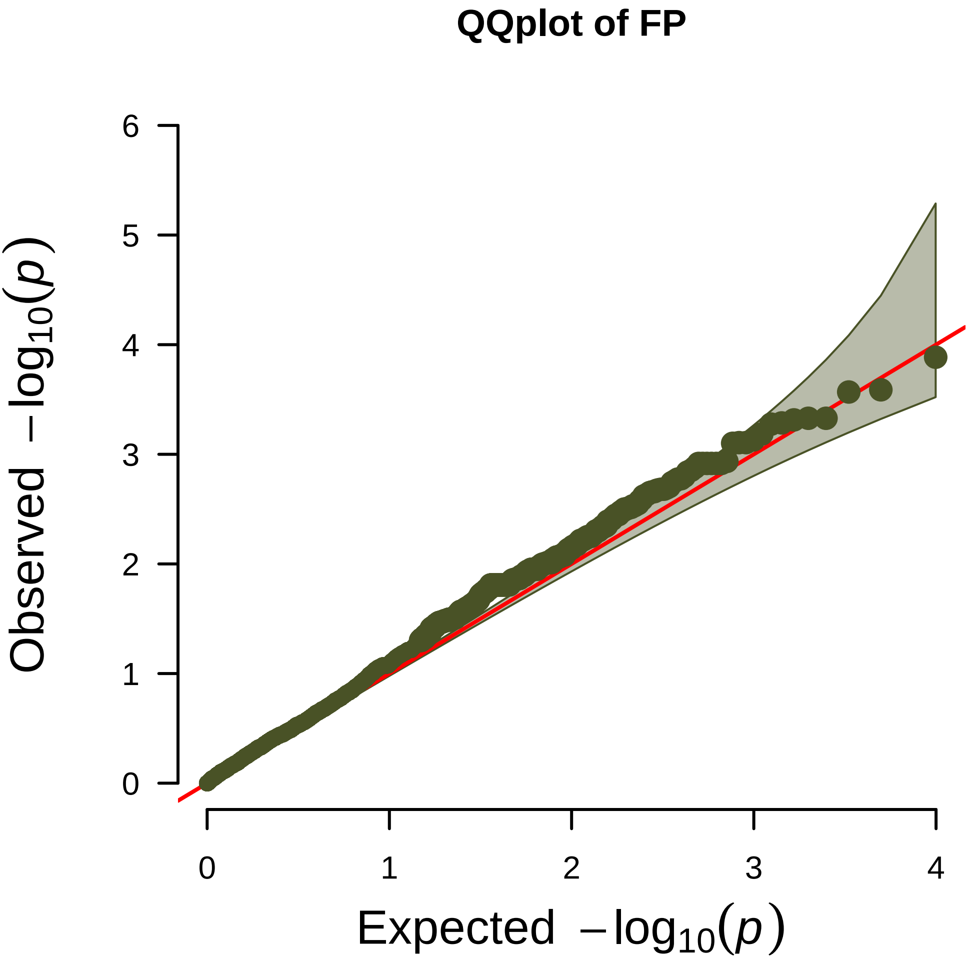

QQplot of FP
Observed −log10(p)
Expected −log10(p)
0
1
2
3
4
5
6
0
1
2
3
4

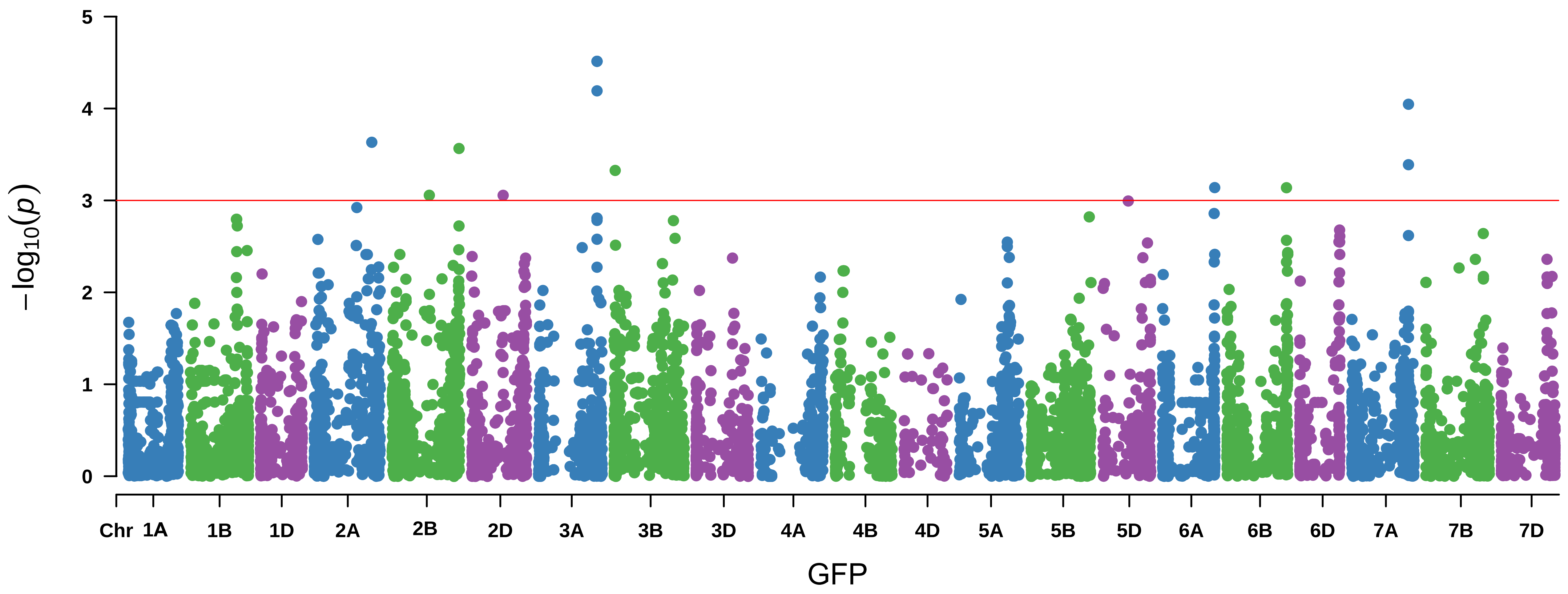

5
4
3
2
1
0
−log10(p)
Chr
1A
1B
1D
2A
2B
2D
3A
3B
3D
4A
4B
4D
5A
5B
5D
6A
6B
6D
7A
7B
7D
GFP

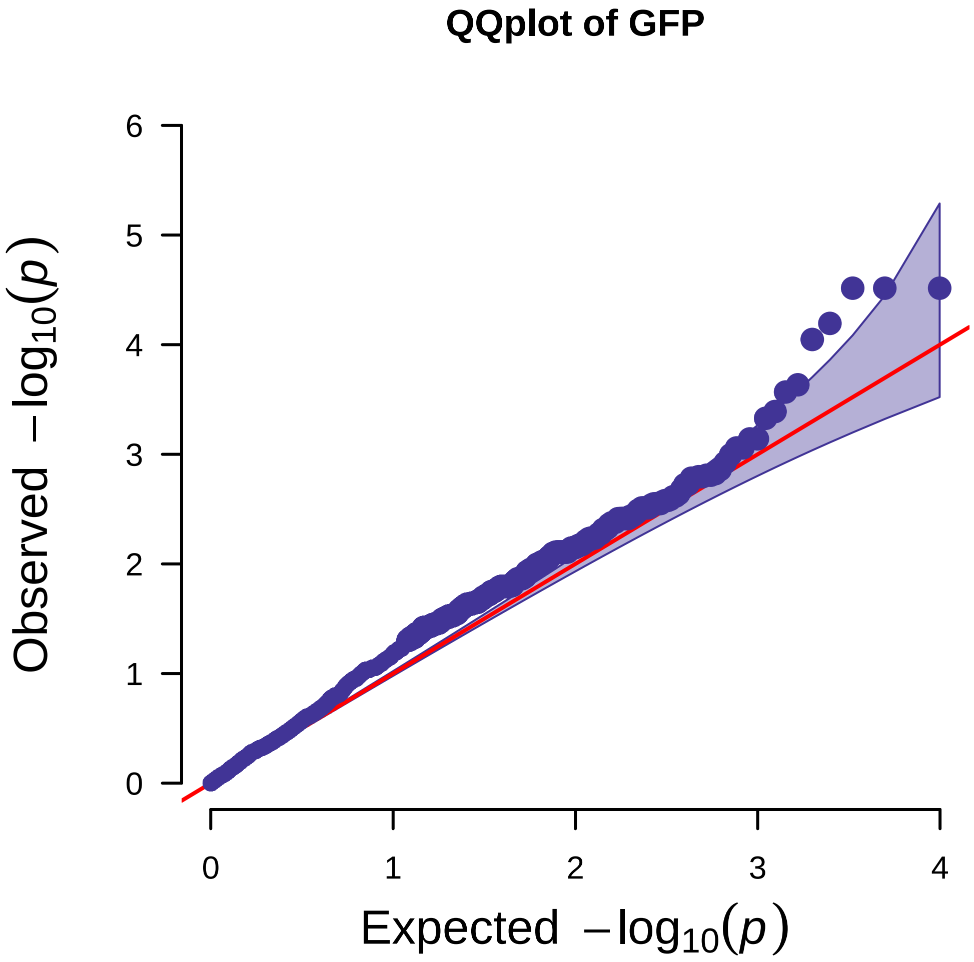
QQplot of GFP
Observed −log10(p)
Expected −log10(p)
0
1
2
3
4
5
6
0
1
2
3
4

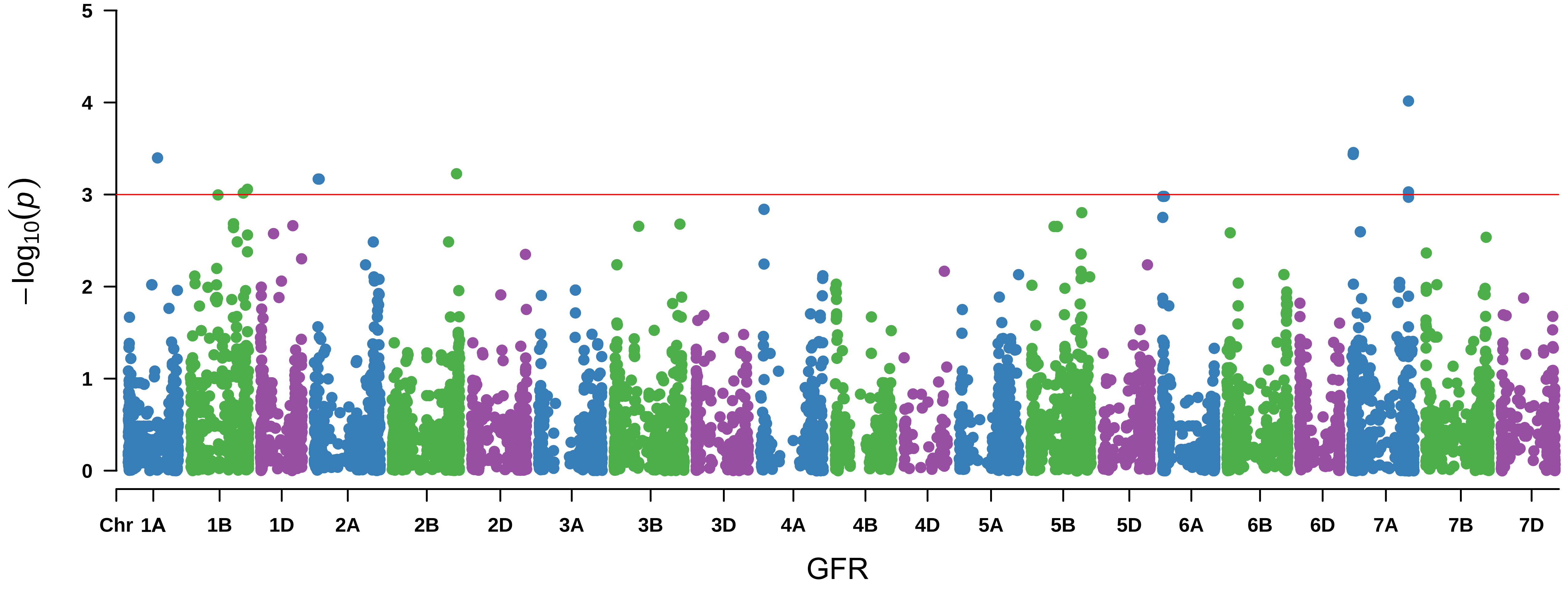

5
4
3
2
1
0
−log10(p)
Chr 1A 1B 1D 2A 2B 2D 3A 3B 3D 4A 4B 4D 5A 5B 5D 6A 6B 6D 7A 7B 7D
GFR

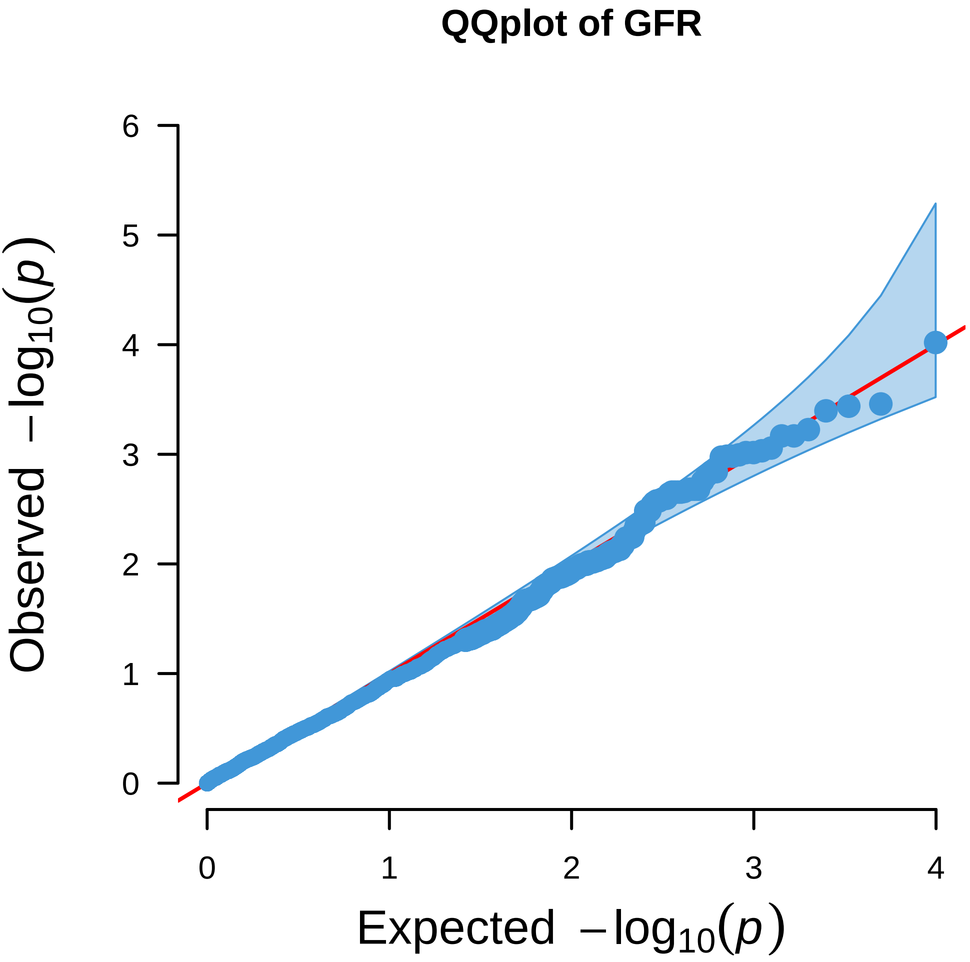
QQplot of GFR
Observed $-\log_{10}(p)$
Expected $-\log_{10}(p)$
0
1
2
3
4
5
6
0
1
2
3
4

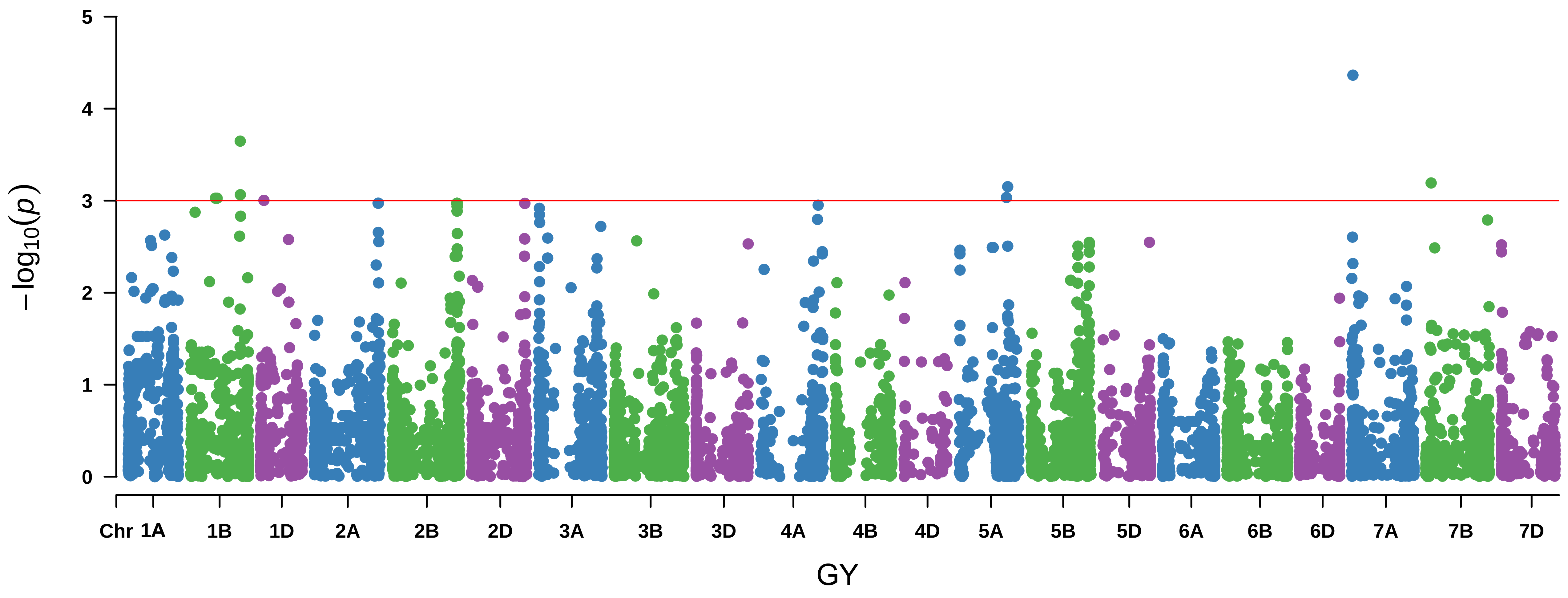

−log10(p)
5
4
3
2
1
0
Chr 1A
1B
1D
2A
2B
2D
3A
3B
3D
4A
4B
4D
5A
5B
5D
6A
6B
6D
7A
7B
7D
GY

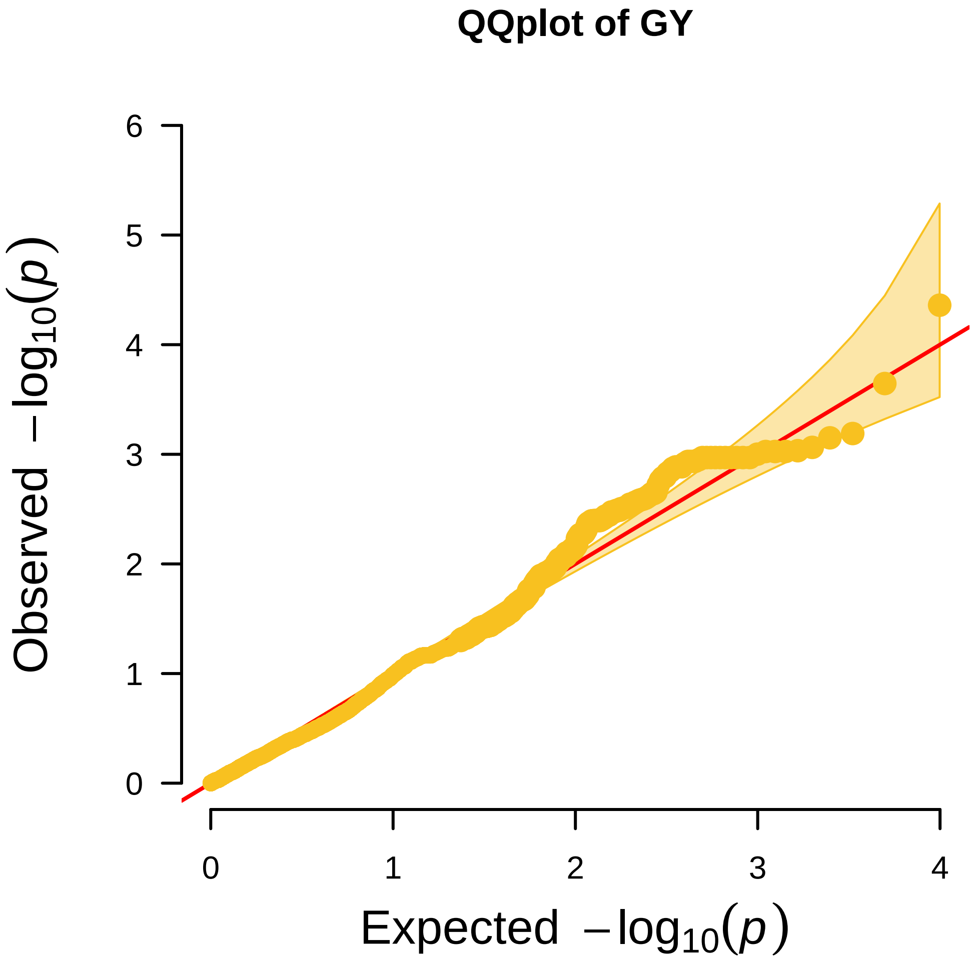
QQplot of GY
Observed $-\log_{10}(p)$
Expected $-\log_{10}(p)$
0
1
2
3
4
5
6
0
1
2
3
4

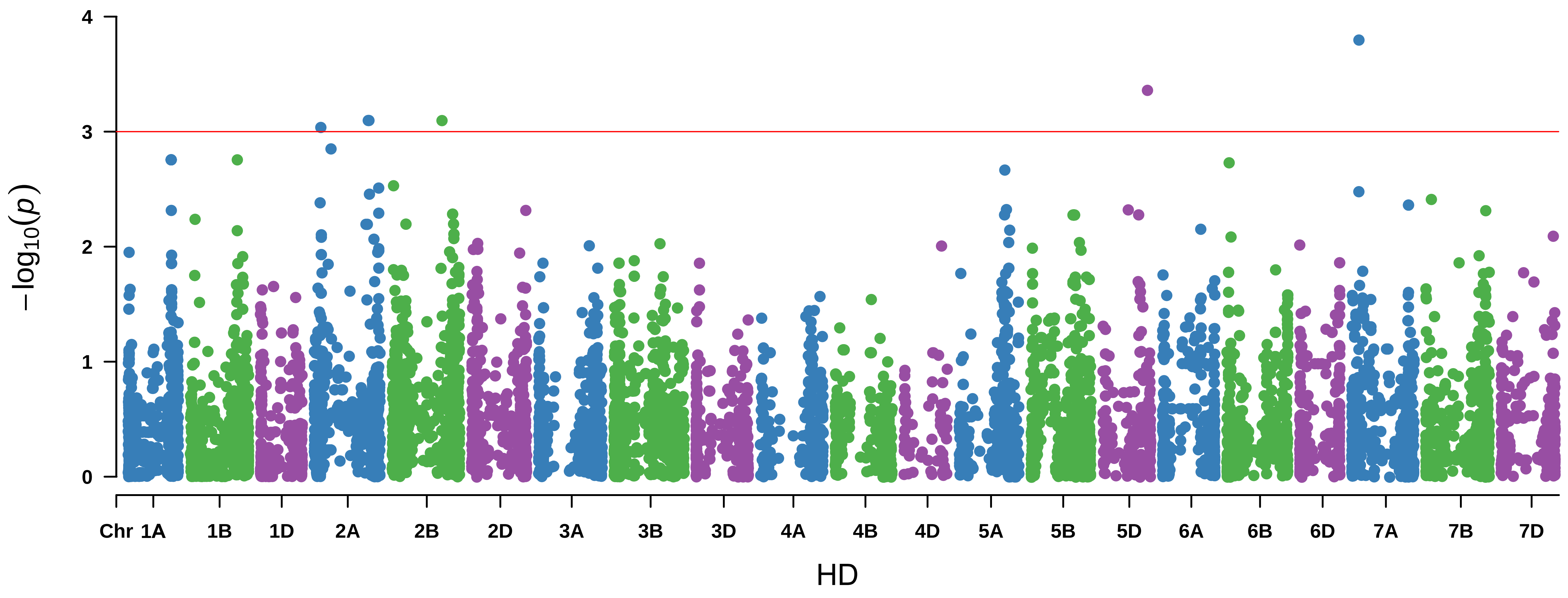

4
3
2
1
0
−log10(p)
Chr 1A 1B 1D 2A 2B 2D 3A 3B 3D 4A 4B 4D 5A 5B 5D 6A 6B 6D 7A 7B 7D
HD

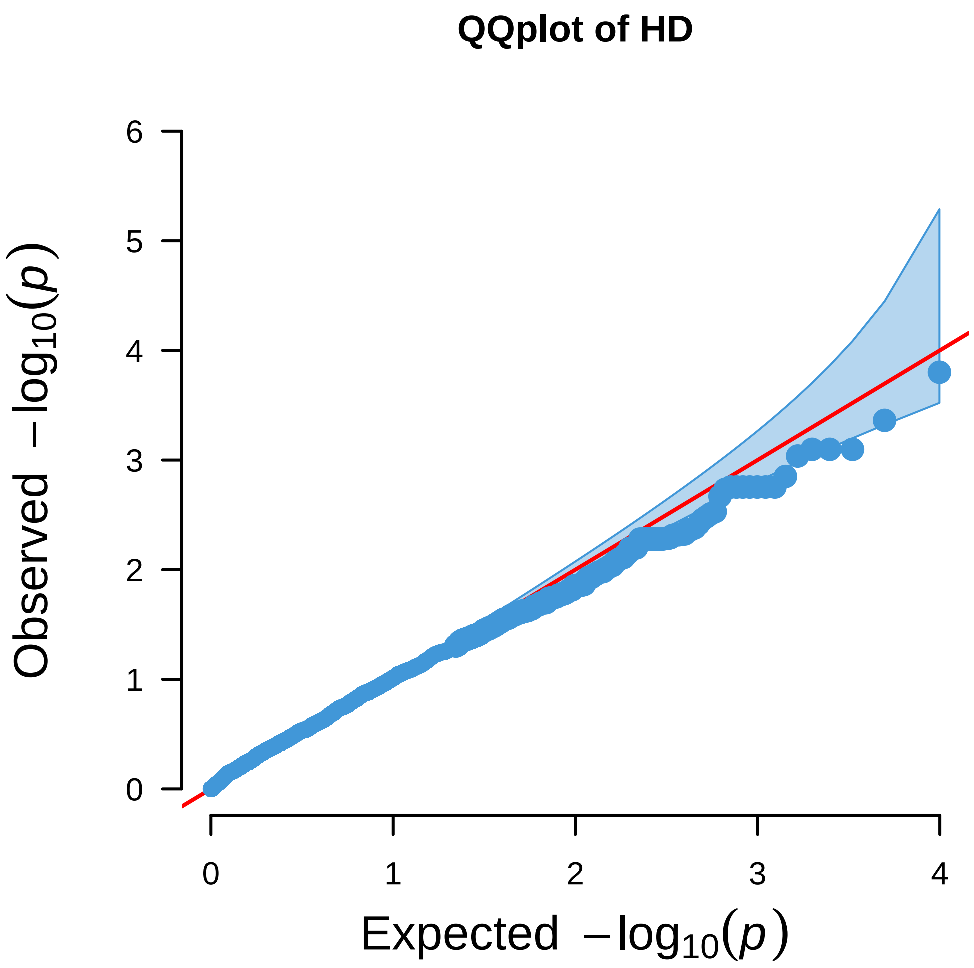
QQplot of HD
Observed −log10(p)
Expected −log10(p)
0
1
2
3
4
5
6
0
1
2
3
4

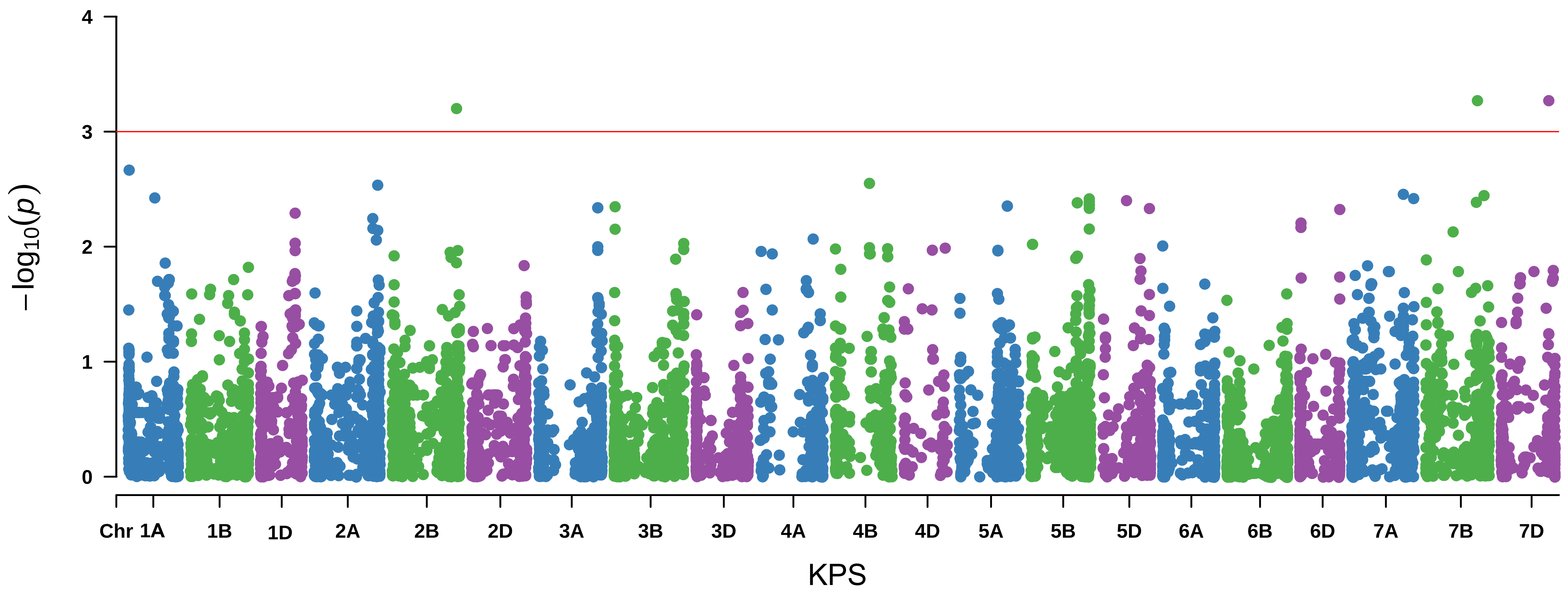

4
3
2
1
0
−log10(p)
Chr 1A
1B
1D
2A
2B
2D
3A
3B
3D
4A
4B
4D
5A
5B
5D
6A
6B
6D
7A
7B
7D
KPS

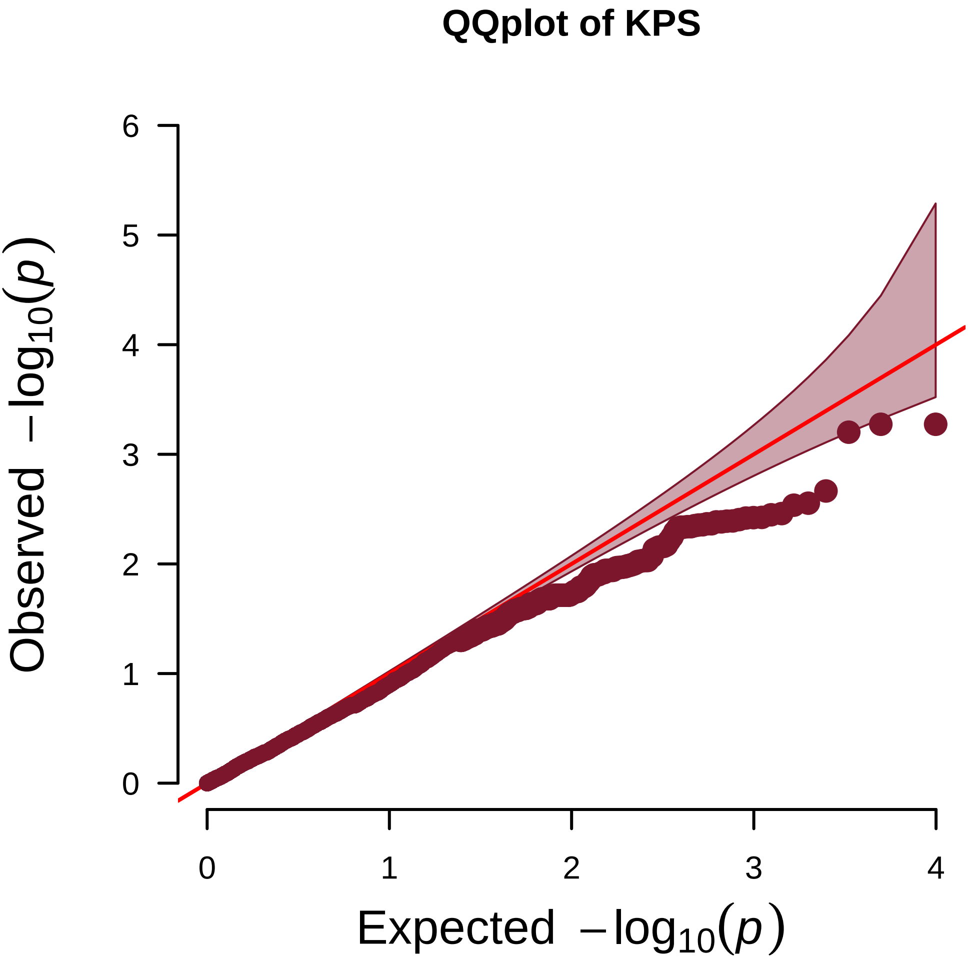
QQplot of KPS
Observed −log10(p)
Expected −log10(p)
0
1
2
3
4
5
6
0
1
2
3
4

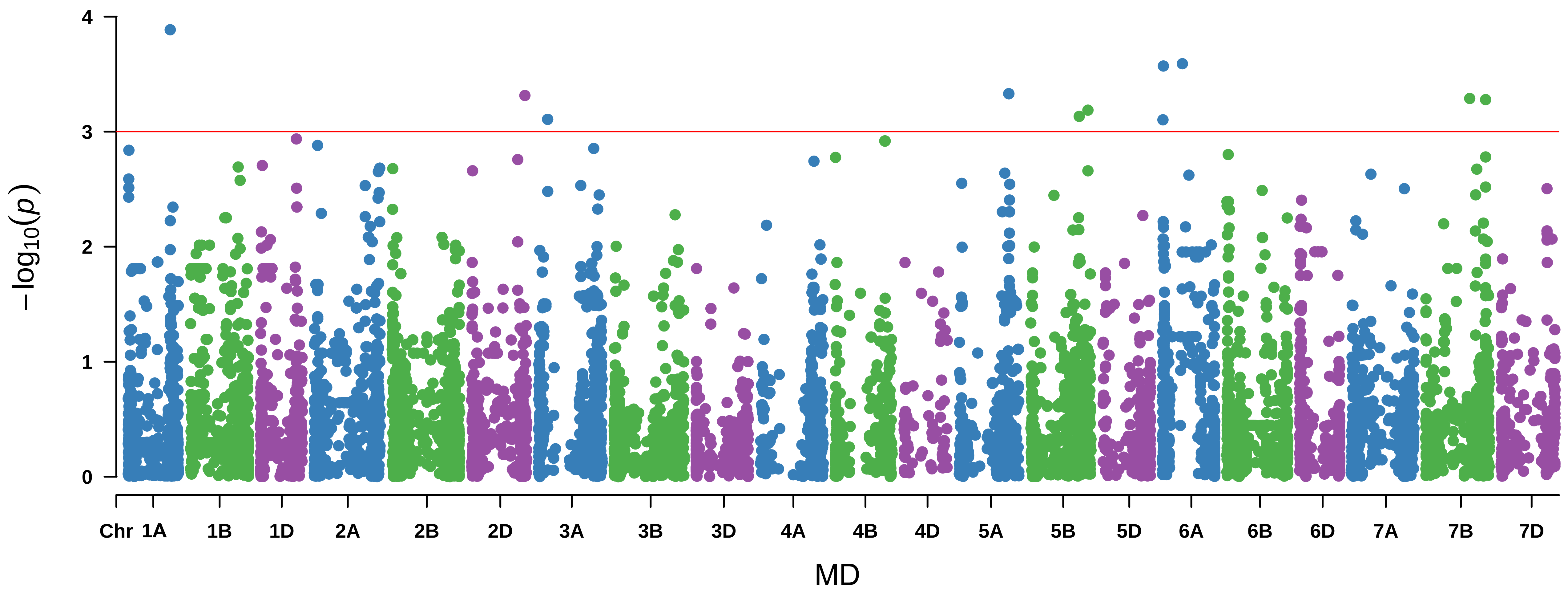

4
3
2
1
0
−log10(p)
Chr
1A
1B
1D
2A
2B
2D
3A
3B
3D
4A
4B
4D
5A
5B
5D
6A
6B
6D
7A
7B
7D
MD

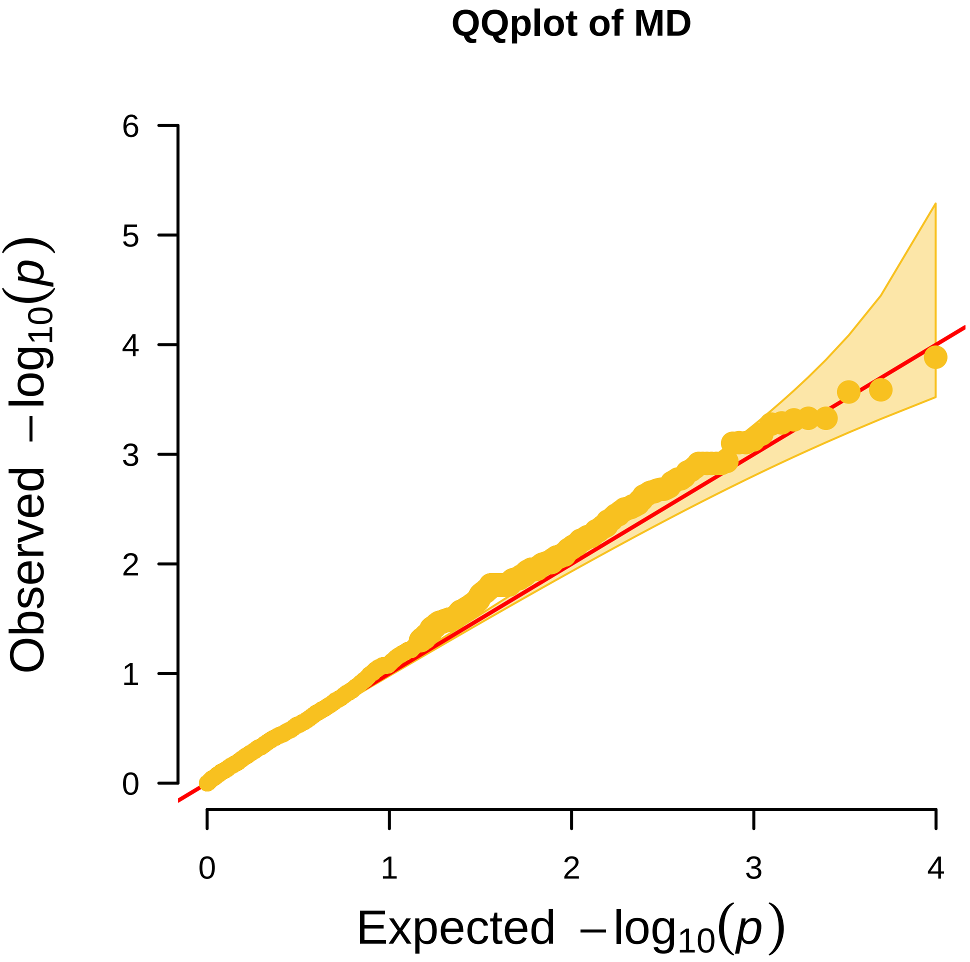
QQplot of MD
Observed −log10(p)
Expected −log10(p)
0
1
2
3
4
5
6
0
1
2
3
4

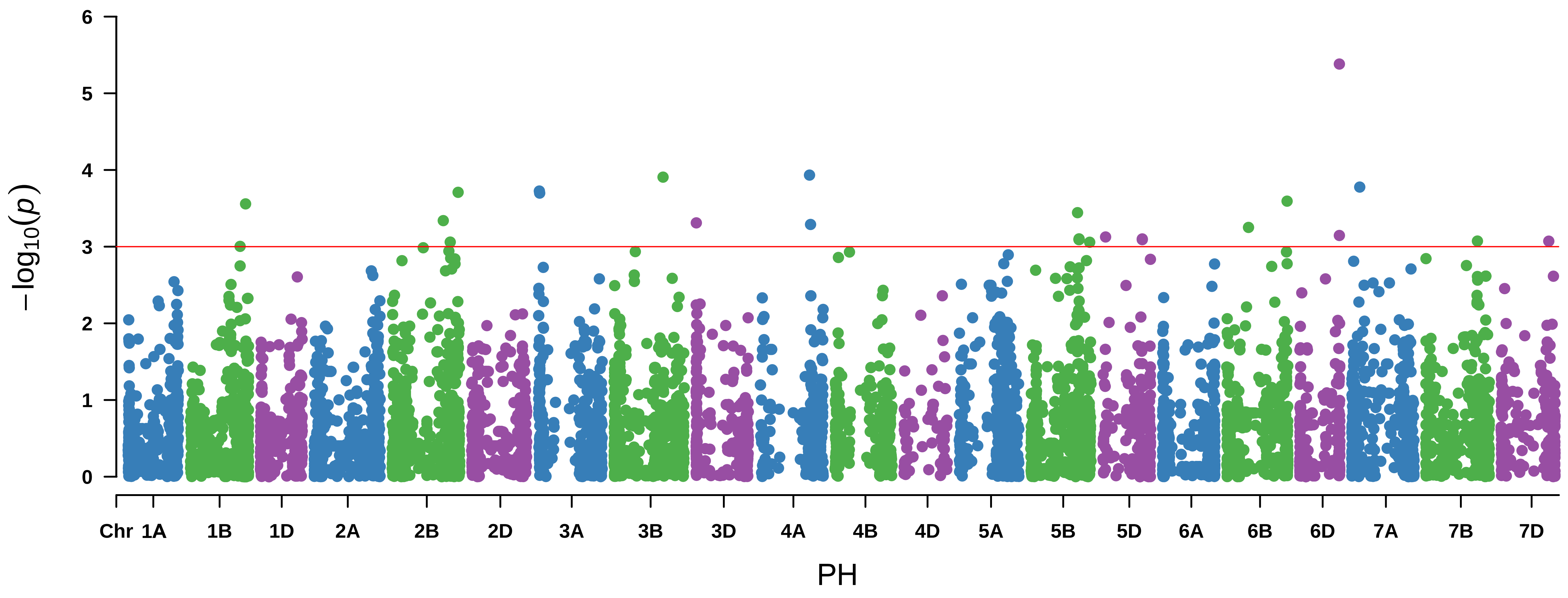

6
5
4
3
2
1
0
$-\log_{10}(p)$
Chr 1A 1B 1D 2A 2B 2D 3A 3B 3D 4A 4B 4D 5A 5B 5D 6A 6B 6D 7A 7B 7D
PH

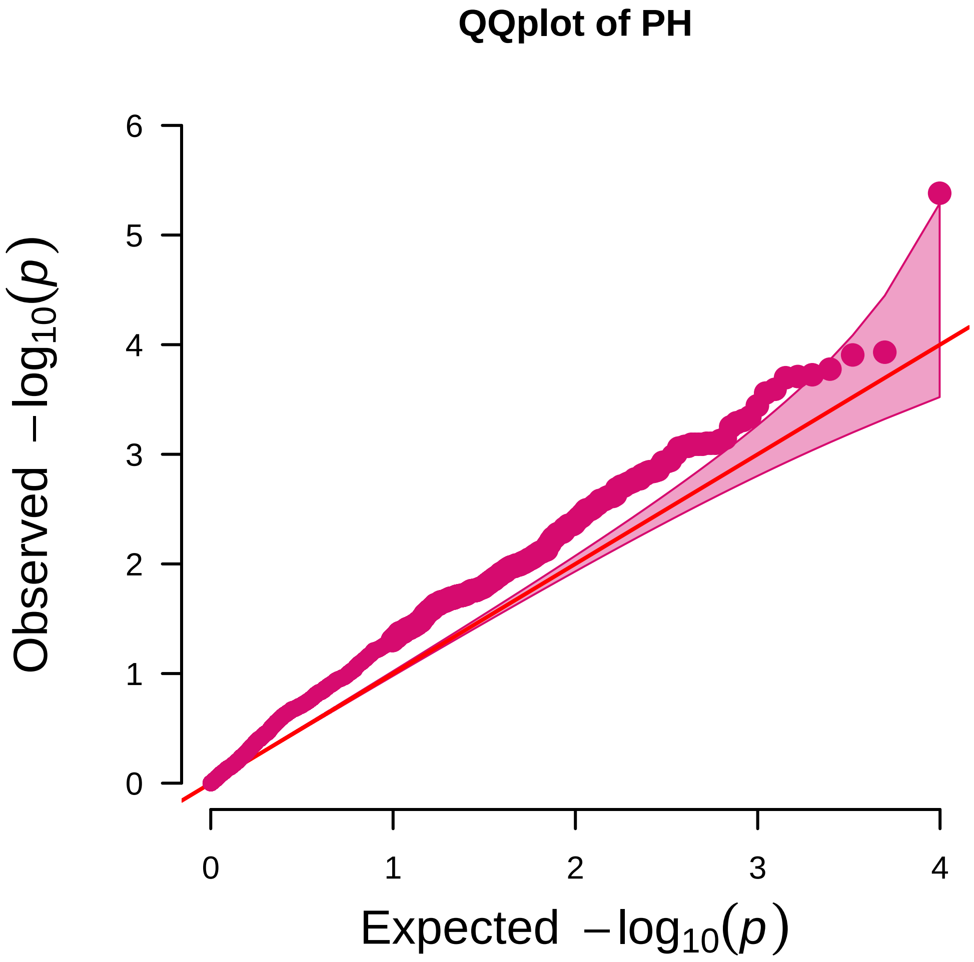
QQplot of PH
Observed −log10(p)
Expected −log10(p)
0
1
2
3
4
5
6
0
1
2
3
4

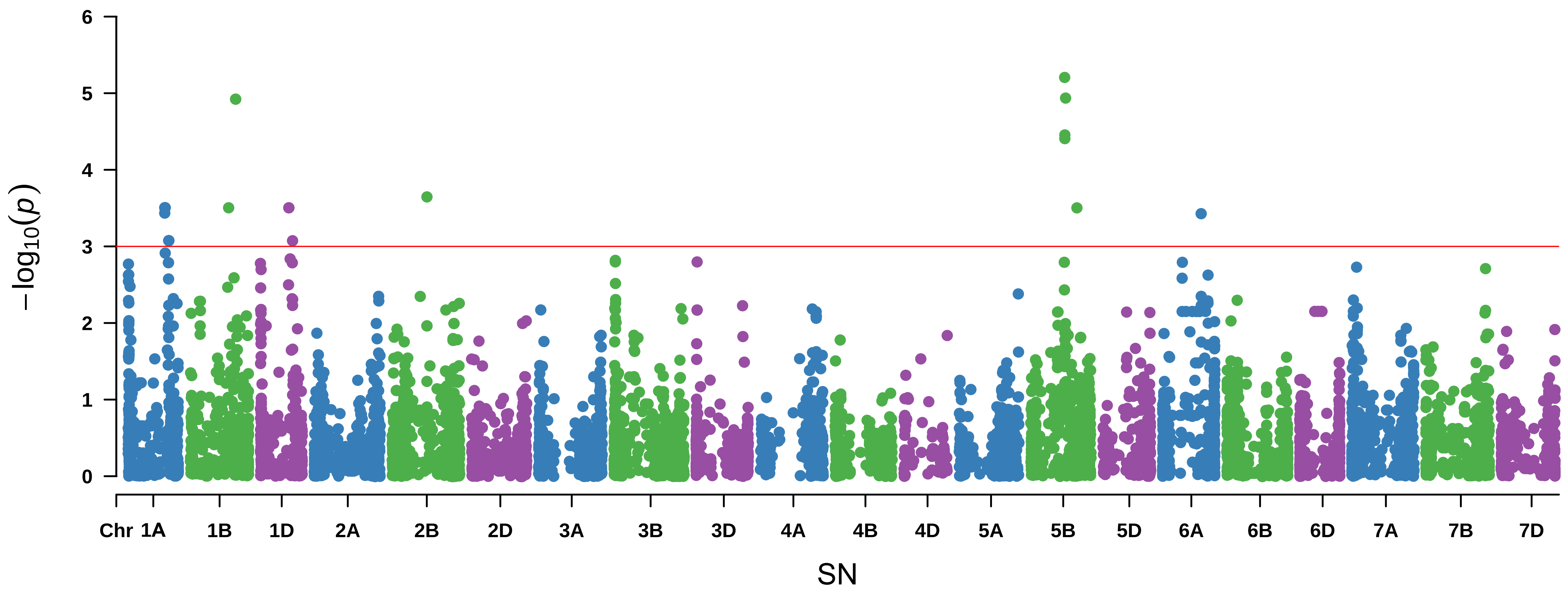

6
5
4
3
2
1
0
−log10(p)
Chr 1A 1B 1D 2A 2B 2D 3A 3B 3D 4A 4B 4D 5A 5B 5D 6A 6B 6D 7A 7B 7D
SN

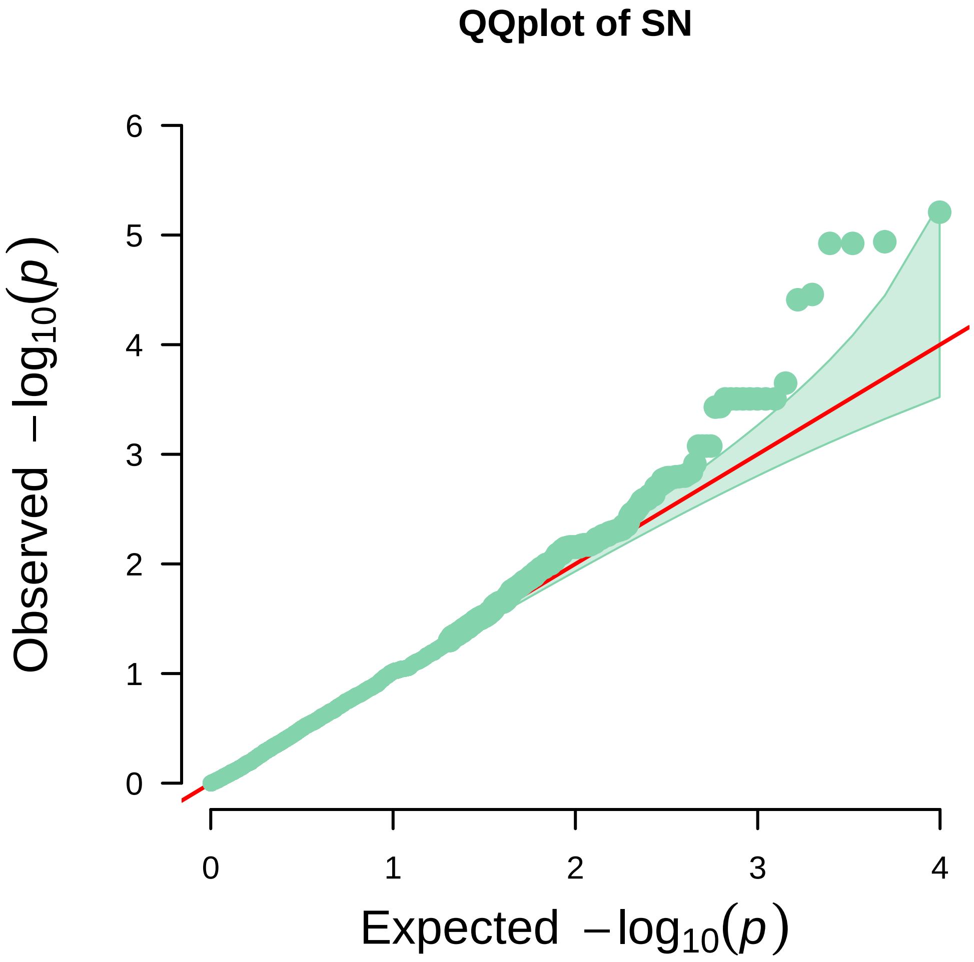
QQplot of SN
Observed $-\log_{10}(p)$
Expected $-\log_{10}(p)$
0
1
2
3
4
5
6
0
1
2
3
4

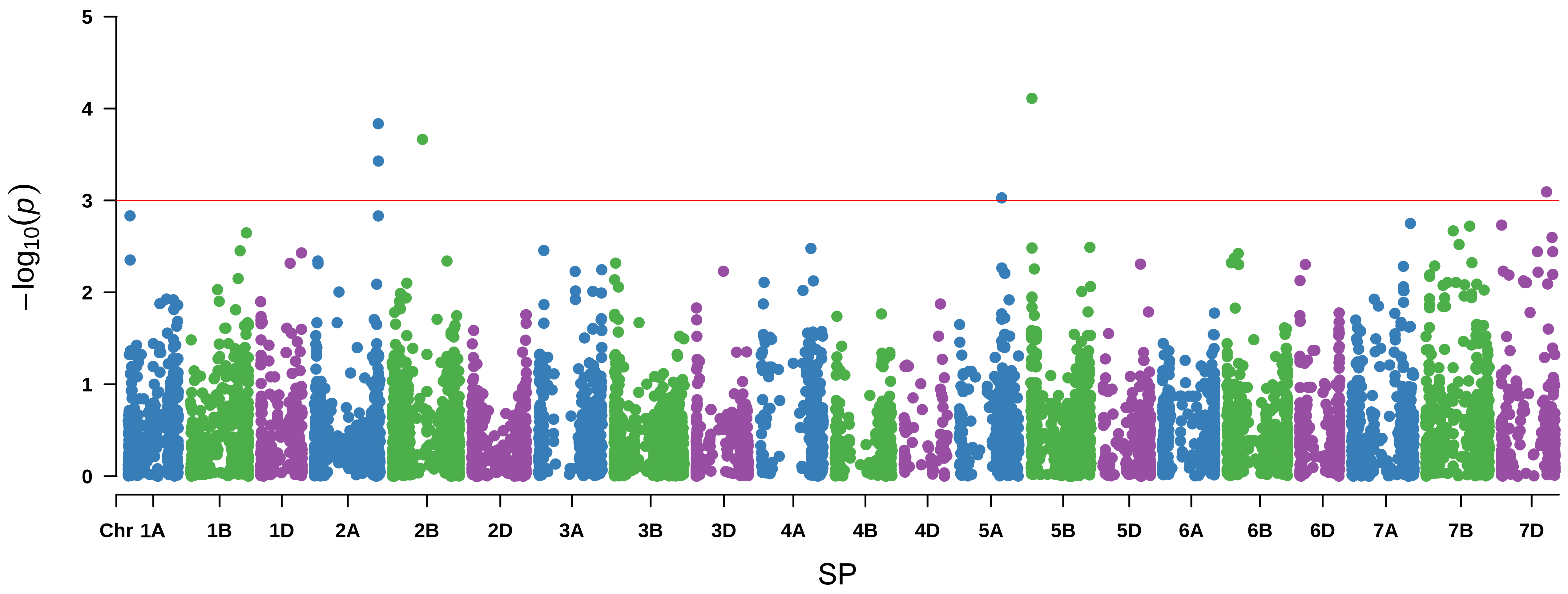

5
4
3
2
1
0
−log10(p)
Chr 1A
1B
1D
2A
2B
2D
3A
3B
3D
4A
4B
4D
5A
5B
5D
6A
6B
6D
7A
7B
7D
SP

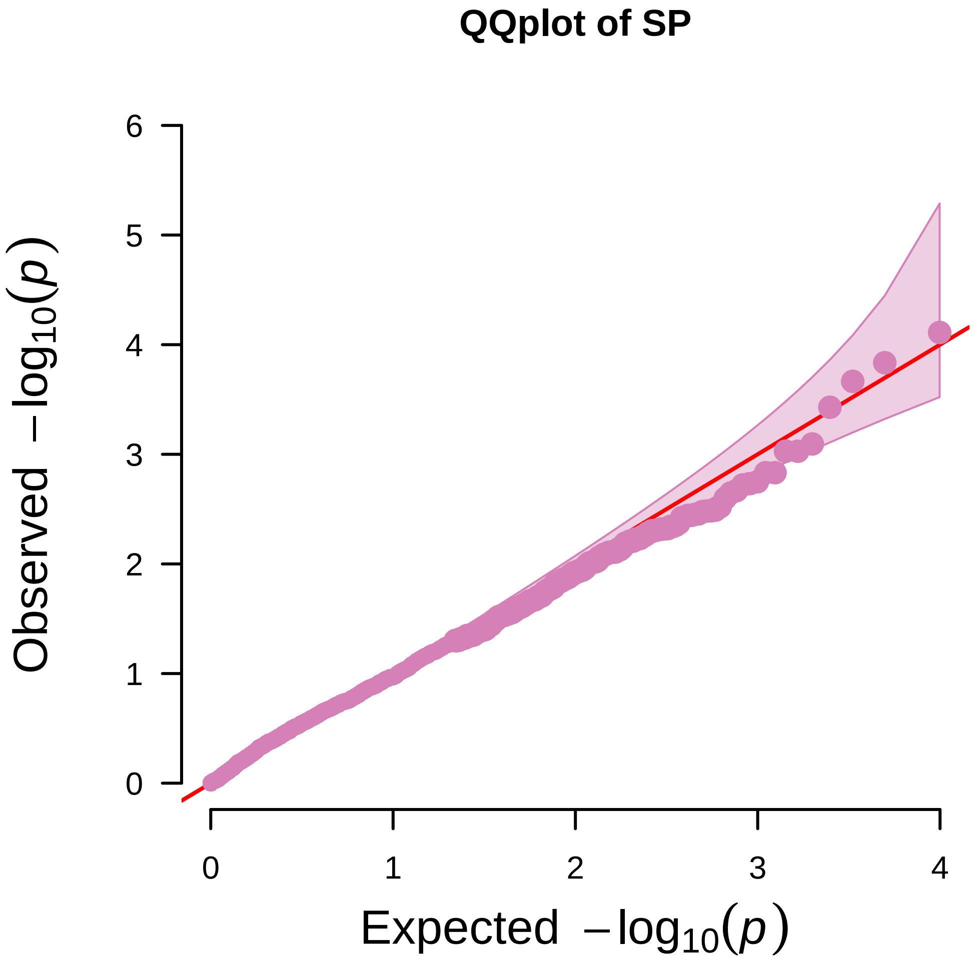
QQplot of SP
Observed $-\log_{10}(p)$
Expected $-\log_{10}(p)$
0
1
2
3
4
5
6
0
1
2
3
4

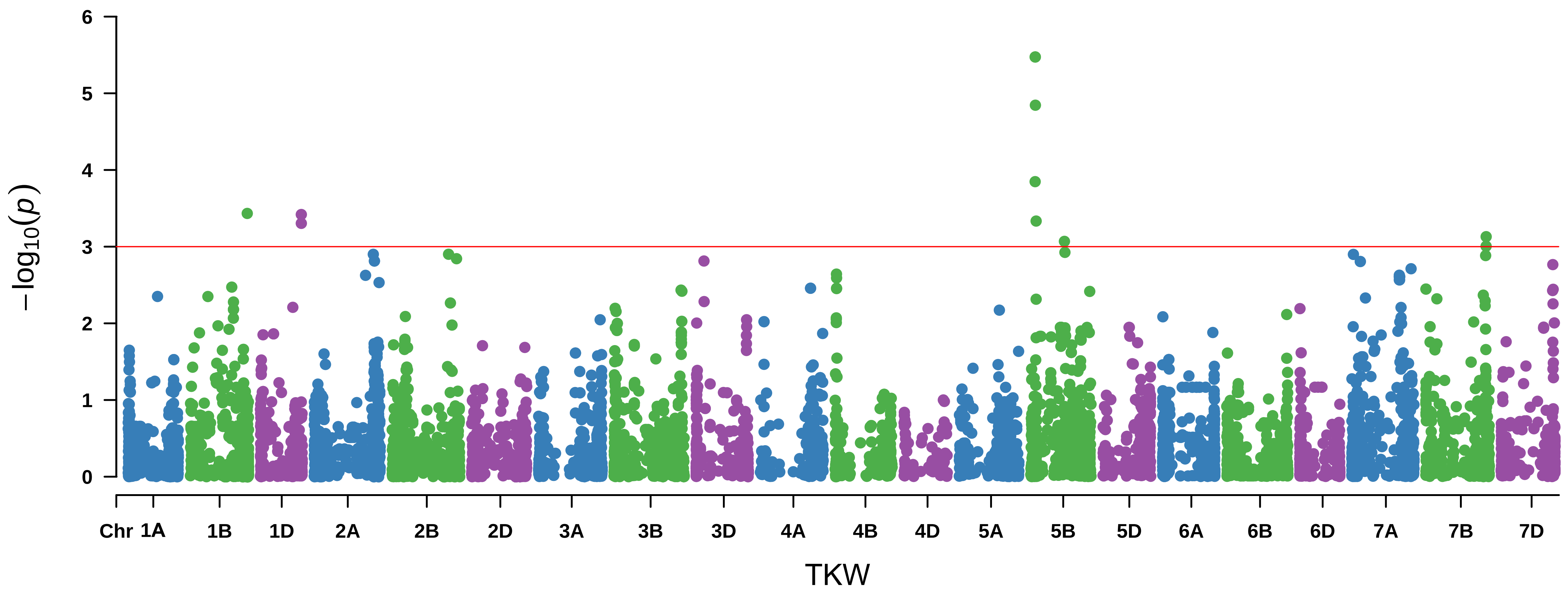

6
5
4
3
2
1
0
−log10(p)
Chr 1A 1B 1D 2A 2B 2D 3A 3B 3D 4A 4B 4D 5A 5B 5D 6A 6B 6D 7A 7B 7D
TKW

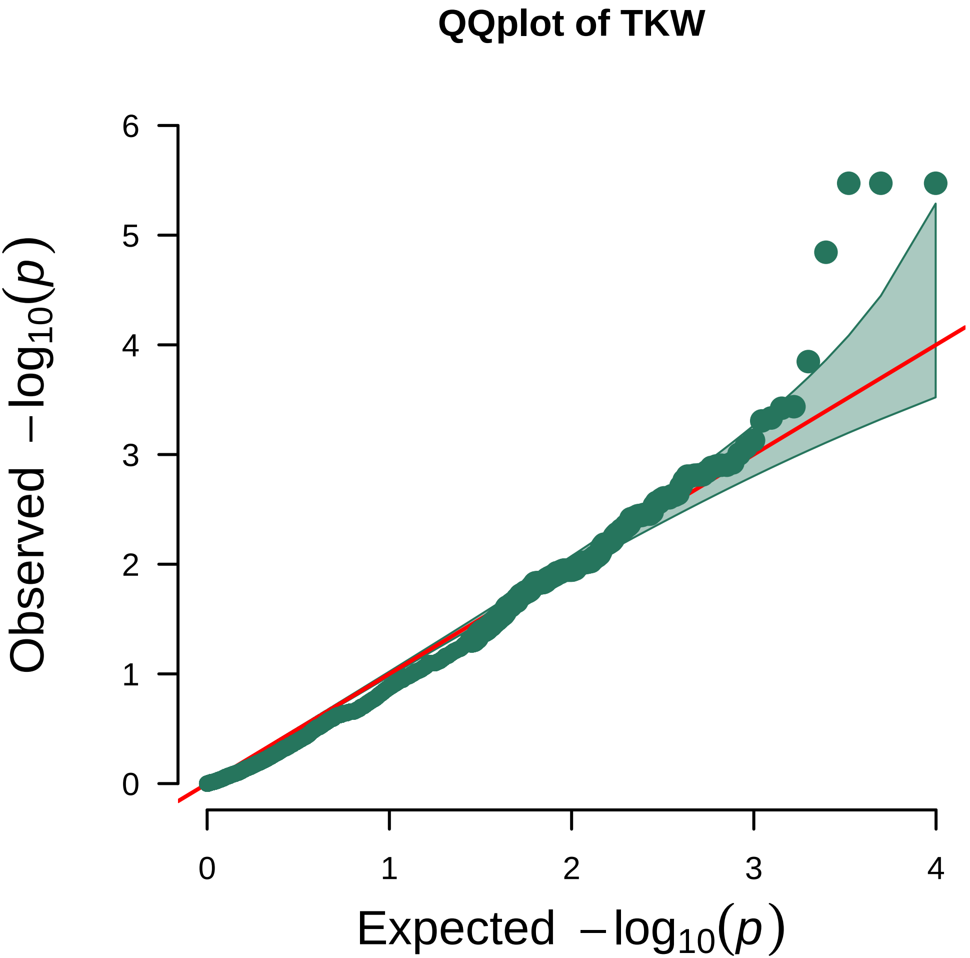
QQplot of TKW
0
1
2
3
4
5
6
Observed $-\log_{10}(p)$
0
1
2
3
4
Expected $-\log_{10}(p)$

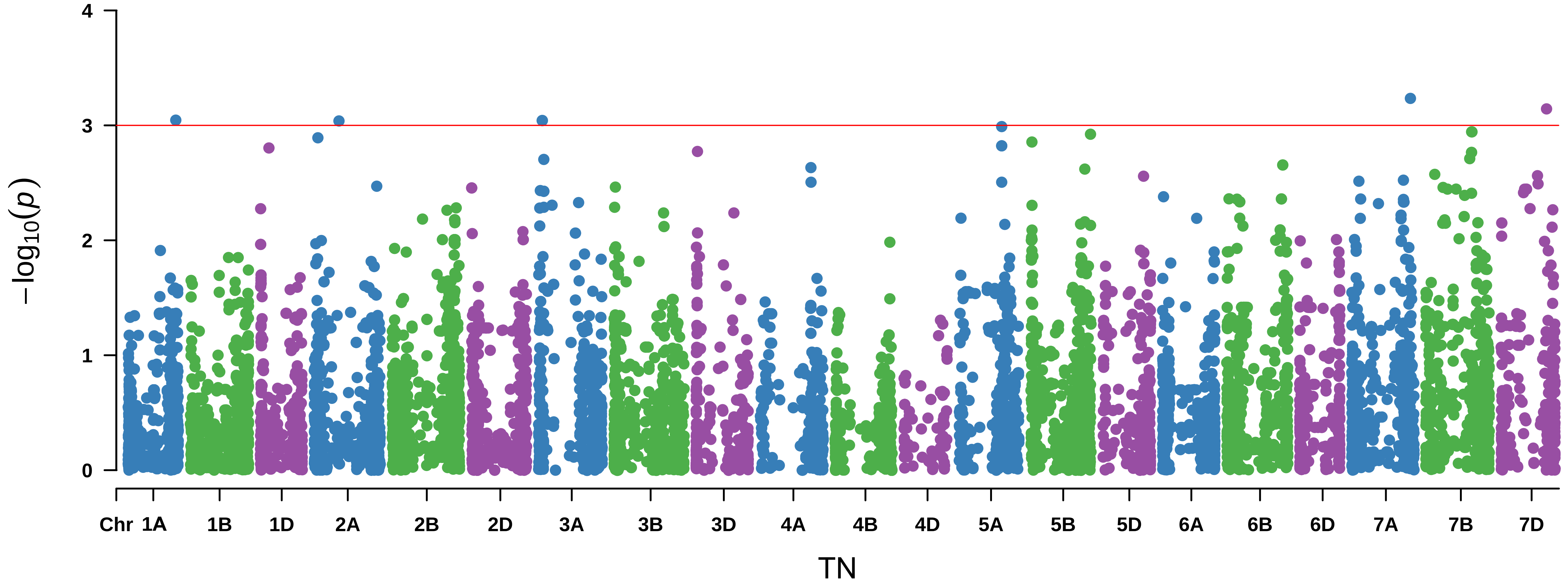

4
3
2
1
0
−log10(p)
Chr
1A
1B
1D
2A
2B
2D
3A
3B
3D
4A
4B
4D
5A
5B
5D
6A
6B
6D
7A
7B
7D
TN

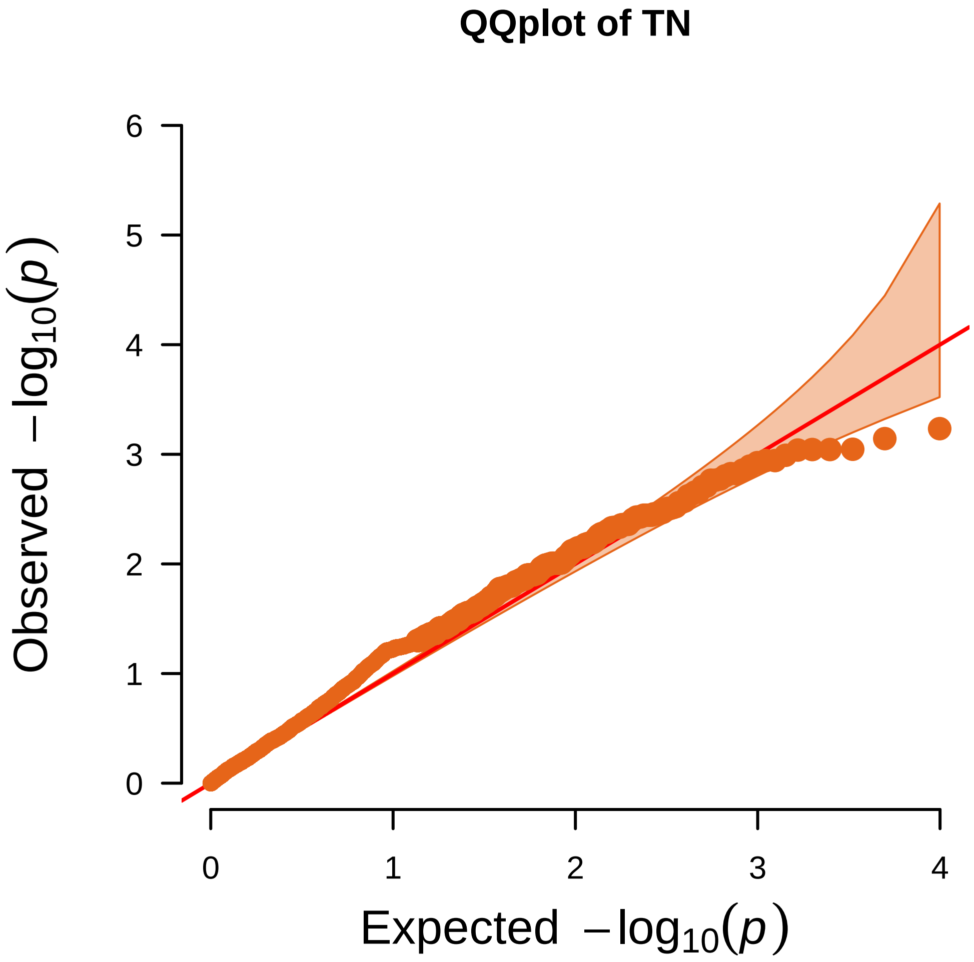
QQplot of TN
Observed $-\log_{10}(p)$
Expected $-\log_{10}(p)$
0
1
2
3
4
5
6
0
1
2
3
4
